# Adverse maternal, fetal, and newborn outcomes among pregnant women with SARS-CoV-2 infection: an individual participant data meta-analysis

**DOI:** 10.1136/bmjgh-2022-009495

**Published:** 2023-01-03

**Authors:** Emily R Smith, Erin Oakley, Gargi Wable Grandner, Kacey Ferguson, Fouzia Farooq, Yalda Afshar, Mia Ahlberg, Homa Ahmadzia, Victor Akelo, Grace Aldrovandi, Beth A Tippett Barr, Elisa Bevilacqua, Justin S Brandt, Nathalie Broutet, Irene Fernández Buhigas, Jorge Carrillo, Rebecca Clifton, Jeanne Conry, Erich Cosmi, Fatima Crispi, Francesca Crovetto, Camille Delgado-López, Hema Divakar, Amanda J Driscoll, Guillaume Favre, Valerie J Flaherman, Chris Gale, Maria M Gil, Sami L Gottlieb, Eduard Gratacós, Olivia Hernandez, Stephanie Jones, Erkan Kalafat, Sammy Khagayi, Marian Knight, Karen Kotloff, Antonio Lanzone, Kirsty Le Doare, Christoph Lees, Ethan Litman, Erica M Lokken, Valentina Laurita Longo, Shabir A Madhi, Laura A Magee, Raigam Jafet Martinez-Portilla, Elizabeth M McClure, Tori D Metz, Emily S Miller, Deborah Money, Sakita Moungmaithong, Edward Mullins, Jean B Nachega, Marta C Nunes, Dickens Onyango, Alice Panchaud, Liona C Poon, Daniel Raiten, Lesley Regan, Gordon Rukundo, Daljit Sahota, Allie Sakowicz, Jose Sanin-Blair, Jonas Söderling, Olof Stephansson, Marleen Temmerman, Anna Thorson, Jorge E Tolosa, Julia Townson, Miguel Valencia-Prado, Silvia Visentin, Peter von Dadelszen, Kristina Adams Waldorf, Clare Whitehead, Murat Yassa, Jim M Tielsch, and Perinatal COVID PMA Study Collaborators, Eduard Langenegger

**Affiliations:** 1 Department of Global Health, The George Washington University Milken Institute School of Public Health, Washington, DC, USA; 2 Division of Maternal Fetal Medicine, University of California Los Angeles, Los Angeles, California, USA; 3 Department of Medicine, Solna, Clinical Epidemiology Division, Karolinska Institute, Stockholm, Sweden; 4 Division of Maternal-Fetal Medicine, The George Washington University School of Medicine and Health Sciences, Washington, DC, USA; 5 Office of the Director, US Centers for Disease Control and Prevention, Kisumu, Kenya; 6 Department of Pediatrics, University of California Los Angeles, Los Angeles, California, USA; 7 Department of Women and Child Health, Women Health Area, Fondazione Policlinico Universitario Agostino Gemelli, IRCCS, Roma, Italy; 8 Division of Maternal-Fetal Medicine, Department of Obstetrics, Gynecology, and Reproductive Sciences, Rutgers Robert Wood Johnson Medical School, New Brunswick, New Jersey, USA; 9 Department of Sexual and Reproductive Health and Research, World Health Organization, Geneve, Switzerland; 10 Department of Obstetrics and Gynecology, Hospital Universitario de Torrejón, Madrid, Spain; 11 Departamento de Obstetricia y Ginecologia, Universidad del Desarrollo Facultad de Medicina Clinica Alemana, Santiago, Chile; 12 The Biostatistics Center, The George Washington University Milken Institute School of Public Health, Rockville, Maryland, USA; 13 International Federation of Gynecology and Obstetrics, London, UK; 14 Department of Women's and Children's Health, University of Padua, Padova, Italy; 15 Department of Maternal-Fetal Medicine, BCNatal, Barcelona Center for Maternal-Fetal and Neonatal Medicine, Hospital Sant Joan de Déu and Hospital Clínic, Universitat de Barcelona, Barcelona, Spain; 16 Surveillance for Emerging Threats to Mothers and Babies, Puerto Rico Department of Health, San Juan, Puerto Rico; 17 Asian Research and Training Institute for Skill Transfer (ARTIST), Bengaluru, India; 18 Center for Vaccine Development, University of Maryland School of Medicine, Baltimore, Maryland, USA; 19 Materno-Fetal and Obstetrics Research Unit, Department ‘Femme-Mère-Enfant’, Lausanne University Hospital, Lausanne, Switzerland; 20 Department of Pediatrics, University of California San Francisco, San Francisco, California, USA; 21 Neonatal Medicine, School of Public Health, Imperial College London Faculty of Medicine, London, UK; 22 Gynecology and Obstetrics, Felix Bulnes Hospital and RedSalud Clinic, Santiago, Chile; 23 South African Medical Research Council, Vaccines and Infectious Diseases Analytics Research Unit, University of the Witwatersrand Faculty of Health Sciences, Johannesburg, South Africa; 24 Department of Obstetrics and Gynecology, Koç University School of Medicine, Istanbul, Turkey; 25 Center for Global Health Research, Kenya Medical Research Institute, Kisumu, Kenya; 26 Nuffield Department of Population Health, University of Oxford, Oxford, UK; 27 Department of Pediatrics, University of Maryland School of Medicine, Baltimore, Maryland, USA; 28 Uganda Virus Institute and the London School of Hygiene & Tropical Medicine, Entebbe, Uganda; 29 Pediatric Infectious Diseases Research Group, St George's University of London, London, UK; 30 Department of Metabolism, Digestion and Reproduction, Imperial College London, London, UK; 31 Department of Obstetrics and Gynecology, University of Washington School of Medicine, Seattle, Washington, USA; 32 Institute of Obstetrics and Gynecology Clinic, Catholic University of Sacred Heart, Rome, Italy; 33 Department of Women and Children’s Health, School of Life Course and Population Sciences, King's College London, London, UK; 34 Clinical Research Division, National Institute of Perinatology, Mexico City, Mexico; 35 RTI International, Research Triangle Park, North Carolina, USA; 36 Departments of Obstetrics and Gynecology, University of Utah Health Sciences Center, Salt Lake, Utah, USA; 37 Division of Maternal-Fetal Medicine, Department of Obstetrics and Gynecology, Northwestern University Feinberg School of Medicine, Chicago, Illinois, USA; 38 Department of Obstetrics and Gynecology, The University of British Columbia, Vancouver, British Columbia, Canada; 39 Department of Obstetrics and Gynecology, The Chinese University of Hong Kong, Hong Kong, Hong Kong; 40 Department of Epidemiology, University of Pittsburgh Graduate School of Public Health, Pittsburgh, Pennsylvania, USA; 41 Kisumu County Department of Health, Kisumu, Kenya; 42 Institute of Primary Health Care (BIHAM), University of Bern, Bern, Switzerland; 43 Pediatric Growth and Nutrition Branch, National Institute of Health, Bethesda, Maryland, USA; 44 Universidad Pontificia Bolivariana, Medellin, Antioquia, Colombia; 45 Centre of Excellence in Women and Child Health, Aga Khan University, Nairobi, Kenya; 46 Department of Obstetrics and Gynecology, St Luke's University Health Network, Bethlehem, Pennsylvania, USA; 47 Centre for Trials Research, Cardiff University, Cardiff, UK; 48 Children with Special Medical Needs Division, Puerto Rico Department of Health, San Juan, Puerto Rico; 49 Department of Women and Children's Health, King's College London Faculty of Life Sciences and Medicine, London, UK; 50 Department of Maternal-Fetal Medicine, The Royal Women's Hospital, University of Melbourne, Parkville, Victoria, Australia; 51 Department of Obstetrics and Gynecology, Sancaktepe Sehit Prof Dr Ilhan Varank Training and Research Hospital, Istanbul, Turkey

**Keywords:** COVID-19, Maternal health, Epidemiology

## Abstract

**Introduction:**

Despite a growing body of research on the risks of SARS-CoV-2 infection during pregnancy, there is continued controversy given heterogeneity in the quality and design of published studies.

**Methods:**

We screened ongoing studies in our sequential, prospective meta-analysis. We pooled individual participant data to estimate the absolute and relative risk (RR) of adverse outcomes among pregnant women with SARS-CoV-2 infection, compared with confirmed negative pregnancies. We evaluated the risk of bias using a modified Newcastle-Ottawa Scale.

**Results:**

We screened 137 studies and included 12 studies in 12 countries involving 13 136 pregnant women.

Pregnant women with SARS-CoV-2 infection—as compared with uninfected pregnant women—were at significantly increased risk of maternal mortality (10 studies; n=1490; RR 7.68, 95% CI 1.70 to 34.61); admission to intensive care unit (8 studies; n=6660; RR 3.81, 95% CI 2.03 to 7.17); receiving mechanical ventilation (7 studies; n=4887; RR 15.23, 95% CI 4.32 to 53.71); receiving any critical care (7 studies; n=4735; RR 5.48, 95% CI 2.57 to 11.72); and being diagnosed with pneumonia (6 studies; n=4573; RR 23.46, 95% CI 3.03 to 181.39) and thromboembolic disease (8 studies; n=5146; RR 5.50, 95% CI 1.12 to 27.12).

Neonates born to women with SARS-CoV-2 infection were more likely to be admitted to a neonatal care unit after birth (7 studies; n=7637; RR 1.86, 95% CI 1.12 to 3.08); be born preterm (7 studies; n=6233; RR 1.71, 95% CI 1.28 to 2.29) or moderately preterm (7 studies; n=6071; RR 2.92, 95% CI 1.88 to 4.54); and to be born low birth weight (12 studies; n=11 930; RR 1.19, 95% CI 1.02 to 1.40). Infection was not linked to stillbirth. Studies were generally at low or moderate risk of bias.

**Conclusions:**

This analysis indicates that SARS-CoV-2 infection at any time during pregnancy increases the risk of maternal death, severe maternal morbidities and neonatal morbidity, but not stillbirth or intrauterine growth restriction. As more data become available, we will update these findings per the published protocol.

**WHAT IS ALREADY KNOWN ON THIS TOPIC**

- Despite the ballooning literature regarding SARS-CoV-2 infection during pregnancy, it is difficult to synthesise the information and evaluate the overall quality of evidence given the heterogeneity in study design, selection of comparison groups, methods for assessing infection, population-specific baseline risks and definitions of key outcomes.
- Prior reviews based on published data have included limited data from low-income countries.

**WHAT THIS STUDY ADDS**

- We established plans for a sequential, prospective meta-analysis in April 2020 with a goal of better understanding the excess risks—or lack thereof—of COVID-19 during pregnancy.
- This individual patient data meta-analysis of unpublished and published data from a dozen studies includes more than 13 000 pregnant women and shows that COVID-19 during pregnancy increases the risk of maternal mortality, intensive care unit admission, receiving mechanical ventilation, receiving any critical care or being diagnosed with pneumonia or thromboembolic disease.
- Infants born to infected pregnant women were more likely to be admitted to the neonatal intensive care unit and to be born premature.
- In contrast to other reviews, we did not find any link between SARS-CoV-2 infection during pregnancy and an increased risk of stillbirth at or beyond 28 weeks’ gestation, nor any link with intrauterine growth restriction.
- Further, we include the first large set of pregnancy cohort data from sub-Saharan Africa.

**HOW THIS STUDY MIGHT AFFECT RESEARCH, PRACTICE OR POLICY**

- Global guidance has been equivocal on the potential risks of infection and benefits and safety of vaccination, and more than 80 countries do not currently recommend that all pregnant and lactating women should be vaccinated.
- Given the clear and consistent findings regarding the risk of COVID-19 infection during pregnancy, global effort to improve access to safe preventives and therapeutics is an urgent priority.

## Introduction

Since early in the pandemic, a key question has been how SARS-CoV-2 infection affects pregnant women and pregnant people, given the physiological, immunomodulatory and mechanical changes that occur during pregnancy. A living systematic review published in February 2021 identified 47 studies comparing pregnant women with COVID-19 versus a contemporaneous or historical group of pregnant women without the disease.^1^ The meta-analysis suggested COVID-19 during pregnancy is linked to increased risk of mortality, intensive care unit (ICU) admission, preterm birth, stillbirth and neonatal care unit admission.^1^ However, for most maternal, fetal and newborn outcomes examined, there were fewer than 10 studies available to synthesise.

More recent electronic healthcare record studies from the USA and a multicountry cohort study found that pregnant women with SARS-CoV-2 infection had higher risks than uninfected pregnant women for pre-eclampsia, eclampsia, caesarean section, ICU admission, stillbirth, preterm birth and neonatal intensive care unit (NICU) admission.^2–4^ A recent population cohort study in England has also linked infection at the time of birth to prolonged hospital stay, often requiring critical care for both mothers and neonates.^5^ Evidence regarding other outcomes such as neonatal mortality, as well as linkages between maternal and child health outcomes, and any potential differences between symptomatic and asymptomatic infections, is limited.^6 7^

Despite the ballooning literature regarding SARS-CoV-2 infection during pregnancy, it is difficult to synthesise the information and evaluate the overall quality of evidence given the heterogeneity in study design, selection of comparison groups, methods for assessing infection, population-specific baseline risks and definitions of key maternal and child health outcomes.^8^ Studies using a universal screening approach to identify SARS-CoV-2 infections are likely to have a higher proportion of asymptomatic or mild cases, and a Swedish study demonstrated that estimates based on non-universal screening data are indeed inflated as compared with universal screening estimates.^6^ Using a ‘not positive’ comparison group results in exposure misclassification and related bias. Globally, key health outcomes such as stillbirth have various definitions, and the published literature does not report on a comprehensive set of maternal and newborn outcomes.

A unified, collaborative analytical plan is required to overcome many of these issues. Accordingly, we established plans for a sequential, prospective meta-analysis (sPMA) in April 2020 with a goal of better understanding the excess risks—or lack thereof—of COVID-19 during pregnancy.^8^ These basic epidemiological data are necessary for conducting appropriate risk-benefit analyses when new preventives and therapeutics are developed and ultimately for guiding global prevention and treatment plans. Our consortium obtained high-quality data from studies being conducted in a variety of countries and analysed them based on a harmonised data collection and analytical strategy. Here, we report the first set of results in this individual participant data (IPD) meta-analysis. We assessed the risk of maternal, fetal and neonatal morbidity and mortality among pregnant women with confirmed or probable SARS-CoV-2 infection during pregnancy as compared with pregnant women who were confirmed SARS-CoV-2 negative.

## Methods

This analysis is part of a larger sPMA study that aims to answer epidemiological questions about COVID-19 and its association with maternal and newborn health by pooling data from independent studies using harmonised data definitions and an IPD meta-analytical framework to minimise data variability. The protocol for the sPMA was registered with PROSPERO (ID: CRD42020188955) on 28 May 2020; the full protocol has been published elsewhere.^8^

### Eligibility criteria

Eligible study designs included registries, single or multisite cohorts, or case–control studies enrolling pregnant women with suspected or confirmed COVID-19. To be eligible, studies must have had a defined catchment area, included at least 25 pregnant women with confirmed or suspected SARS-COV-2 infection and had a contemporaneously recruited comparison group of pregnant women who had not been diagnosed with COVID-19.

Given the heterogeneity of study designs, we also applied participant-level inclusion and exclusion criteria. The SARS-CoV-2 infected group included pregnant women with a diagnosis during pregnancy or within 7 days of pregnancy outcome based on: (a) PCR testing or antigen testing; (b) WHO suspected case definition^9^; or (c) serology testing where exposure was known to occur during pregnancy based on the dates of the pregnancy and the COVID-19 pandemic. We restricted the analyses to a comparison group of pregnant women who were confirmed SARS-CoV-2 negative based on one or more laboratory tests for SARS-CoV-2 infection during pregnancy (including PCR, antigen or serology testing).

### Identifying studies

For this comparative analysis, we identified studies using two approaches. Studies were recruited into the sPMA via professional research networks and support from key stakeholder networks a priori,^8^ and those who had agreed to participate by 1 August 2020 were screened for eligibility to participate in this analysis. We also identified studies by reviewing the most recently published (February 2021) PregCOV-19 Living Systematic Review^1^ to identify studies that might be eligible for postpublication inclusion into the analysis; we contacted all corresponding authors of apparently eligible studies. Studies were first screened for eligibility based on published protocols or manuscripts; we also confirmed eligibility through discussions with study investigators.

### Data collection

Data contributors shared deidentified IPD with the sPMA coordinating team based on a core variable list.^8^ The coordinating team ran a standardised set of data quality codes and resolved any queries through discussion with the study investigators. Subsequently, we created new, harmonised outcome variables and analysed the data to ensure consistent methods were used to generate site-specific estimates. Study investigators reviewed these estimates. Where data contributors were unable to share IPD, the coordinating team worked with the contributing statistical team to use the same set of standardised outcome definitions and/or codes for data quality assessment, outcome construction and generating site-specific estimates; these teams shared analysis log files and outputs to confirm the same analysis process was followed. We checked each data set for potentially overlapping participants based on the geographic area or facility and enrolment dates; we worked with study investigators to deduplicate any potential overlapping observations. For each previously published study, online supplemental table S1 documents reasons for any differences between the data included in this study as compared with prior publications. This secondary use of deidentified data was considered non-human subjects research and thus exempt from institutional review board approval at The George Washington University.

10.1136/bmjgh-2022-009495.supp1 Supplementary data

### Data items

The core variables for the larger sPMA study were established a priori along with the protocol.^8^ For this analysis, the coordinating team developed an analysis plan, which was reviewed and approved by the steering committee. Participating study sites contributed data based on this shortlist of high-priority variables. Based on IPD from each study, we derived each study outcome described below.

### IPD integrity (data quality assessment)

Data quality was assessed for each study by examining the distribution and frequency of each variable. We identified outliers and inconsistent values for key data points such as gestational age at birth, maternal age and neonatal birth weight and checked that the timing of outcomes was consistent with our definitions (eg, neonatal death within 28 days). For all published data, we also compared the distribution and frequency of outcomes to published manuscripts and resolved discrepancies through discussion with study investigators.

### Risk of bias

We assessed the quality of individual studies, by outcome, based on criteria for participant selection and outcome ascertainment using an adapted Newcastle-Ottawa Scale.^10^ A description of study design elements classified as lower or higher risk of bias is outlined in online supplemental table S2.

### Outcomes and effect measures

We considered four categories of outcomes including hospital and critical care indicators, maternal mortality and morbidity, fetal and neonatal mortality and morbidities and adverse birth outcomes. Maternal, fetal and neonatal death and adverse birth outcomes were defined using WHO case definitions. Hospital and critical care indicators and maternal morbidities were defined by each contributing study. *Critical care indicators* included outcomes related to COVID-19 severity: admission to the ICU, receipt of critical care (defined as admitted to ICU or received ventilation or any site-defined indicator), any ventilation use and clinician-diagnosed pneumonia. *Maternal mortality and morbidity outcomes* included maternal death (due to any cause during pregnancy or 42 days post partum),^11^ haemorrhage around the time of labour, placental abruption, hypertensive disorders of pregnancy (diagnosed at or after testing positive for COVID-19), hypertensive disorders of pregnancy (diagnosed at any time), pre-eclampsia, eclampsia, pre-eclampsia or eclampsia (a combined indicator), thromboembolic disease, preterm labour, any caesarean delivery and intrapartum or non-scheduled caesarean delivery. *Fetal and neonatal mortality and morbidity outcomes* included stillbirth (fetal death >28 weeks),^12^ perinatal death (stillbirth >28 weeks or neonatal death in the first 7 days of life),^13^ early neonatal death (death in the first 7 days of life),^14^ neonatal death (death in the first 28 days of life) and admission to the NICU; in one study (Crovetto, 2020), we collected a combined outcome of NICU admission and/or admission to a high-dependency care unit. *Adverse birth outcomes* included combined extremely, very and moderate preterm birth (<34 weeks’ gestational age at birth), preterm birth (<37 weeks’ gestational age at birth), very low birth weight (<1500 g), low birth weight (<2500 g) and small for gestational age (<3rd or <10th percentile of sex-specific size for gestational age based on the INTERGROWTH-21st reference values^15^; for studies without data on infant sex, we used the midpoint of sex-specific percentiles).

### Statistical analysis (synthesis methods)

We applied a two-stage IPD meta-analytical framework (accounting for site-specific clustering) to generate pooled absolute risks and relative risks (RR), along with 95% CIs, for each outcome. First, we estimated site-specific prevalence estimates for the infected and uninfected groups, as well as unadjusted and adjusted RR with 95% CIs. We originally produced unadjusted and adjusted RRs for each site contributing data. We adjusted for maternal age and, where available, pre-pregnancy obesity (pre-pregnancy body mass index (BMI) >30 kg/m^2^). Because we found very little difference in adjusted and unadjusted RRs within each site, we proceeded with the meta-analysis using unadjusted RRs to allow inclusion of studies with zero outcome event in either the exposed or unexposed group. We pooled the absolute risks of each outcome using the Freeman-Tukey double arcsine transformation with DerSimonian and Laird random-effects model; we calculated exact 95% CIs.^16 17^ RRs were pooled using DerSimonian and Laird random-effects meta-analysis.^18^ Heterogeneity was assessed using the I^2^ statistic.

In cases of zero event for an outcome in the exposed or unexposed group, we applied a continuity correction of 0.5. Outcomes with zero event in both arms were omitted when estimating pooled absolute risk and pooled RRs because the infected and uninfected groups varied in size. All participants in a study were excluded from an analysis if more than 25% of participants were missing outcome information.

Not all studies collected information about the date of COVID-19 onset (symptoms or test dates) and the date of each outcome; however, we performed a sensitivity analysis restricting the analysis to those studies with known date of onset as well as dates for three outcomes: preterm labour, preterm birth and moderate preterm birth. For preterm labour and preterm birth outcomes, we restricted the sensitivity analyses to women with gestational age of COVID-19 onset at less than 37 weeks and for moderate preterm birth by restricting the analyses to women with gestational age of onset at less than 34 weeks. For the outcome hypertensive disorders of pregnancy, we conducted a sensitivity analysis looking at diagnoses that occurred at or after COVID-19 diagnosis.

To address concerns about the varying degree to which studies employed universal screening strategies and thus identified asymptomatic pregnant women, we conducted a secondary analysis restricting exclusively to symptomatic cases of COVID-19. Further, we conducted a sensitivity analysis comparing our results to those studies included in the PregCOV-19 Living Systematic Review that were eligible for the PMA but not successfully recruited to examine any major differences in results across seven common outcomes. Finally, we conducted a sensitivity analysis using different definitions of stillbirth to examine differences based on gestational age cut-offs. All analyses were performed using Stata (V.16), SAS (V.9.4) and R (V.4.2.0).

### Patient and public involvement

Patients or the public were not involved in the design, conduct, reporting or dissemination plans of our meta-analysis. However, many contributing studies did involve patients and community stakeholders in the design and dissemination of their study results.

## Results

### Study selection

**Figure 1:**
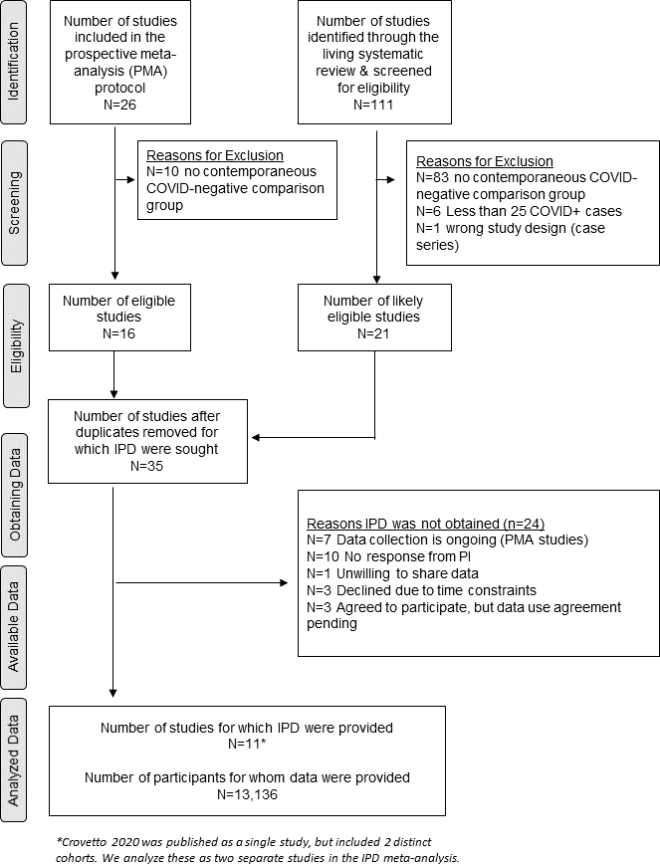
PRISMA-IPD flow diagram documenting study identification, screening and analysis. IPD, individual participant data; PI, principal investigator; PRISMA, Preferred Reporting Items for Systematic Reviews and Meta-Analyses.

Among the 26 studies that had prospectively joined the PMA study team, 16 had a study design that allowed for the comparison of SARS-CoV-2 infected and uninfected pregnancies. Six of these studies had completed data collection or were willing to contribute ongoing cohort data to the current analysis (Akelo and Tippett Barr 2021, Bevilacqua and Laurita Longo 2020, Le Doare 2021, Nachega 2021, Nunes 2021, Poon 2021). We additionally contacted the corresponding authors of apparently eligible studies included in the Allotey *et al*’s living systematic review and identified five additional studies that were willing to participate in this round of the sPMA^19–23^ (figure 1). One of these studies included two different testing strategies for two cohorts of pregnant women (Crovetto, 2020); accordingly, we consider this publication and related data collection as two separate studies.

We identified and deduplicated three participants who were included in both the current AFREhealth (Nachega) and PREPARE Uganda (Le Doare) data sets. No other overlapping participants were identified.

### Study characteristics

In total, we analysed IPD from *12 studies conducted in 12 countries* (Ghana, China-Hong Kong, Italy, Kenya, Nigeria, South Africa, Spain, Sweden, the Democratic Republic of Congo, Turkey, Uganda and the USA) (table 1). Across studies, the recruitment period spanned from February 2020 to July 2021 (online supplemental figure S1).^24 25^ Across all studies, SARS-CoV-2 *infection was confirmed by PCR test*, except in the following studies: Crovetto 2020 Cohort I study administered antibody tests at recruitment in early pregnancy and PCR tests at delivery; Crovetto 2020 Cohort II study used antibody tests at delivery for all participants (and 85% also received a PCR test); Le Doare (2021) used the WHO case definition for probable cases of COVID-19 when testing was unavailable in addition to PCR and antibody testing at recruitment; and Ahlberg *et al* 19 where three cases were identified on admission for delivery based on positive antibody test results during antenatal care (ANC). *Selection of the SARS-CoV-2-negative group* varied slightly between studies; seven studies defined SARS-CoV-2-negative pregnancy based on a single negative PCR test result (Nachega, Nunes, Sakowicz, Ahlberg, Bevilacqua and Laurita Longo, Kalafat, Brandt), one study based the selection on repeated negative PCR tests throughout pregnancy (Akelo and Tippett Barr), two studies used a negative antibody test result (Crovetto, Poon) and one population-based pregnancy surveillance study ascertained SARS-CoV-2 infection using PCR and/or antibody testing at recruitment, followed by testing or assessment for probable diagnosis based on clinical concern (Le Doare). The timing of testing varied by study, but most studies included infections in all three trimesters (table 2).

**Table 1:** Study characteristics

Data source	Study	Location	Dates of data collection	Recruitment strategy*					COVID-19 case definition	COVID-negative comparison group
Prospective pregnancy cohort studies	Akelo and Tippett Barr (2021)	Kenya	July 2020 to May 2021, with follow-up through delivery	a	b	c	d	e	Positive PCR test	Negative PCR test
	Le Doare (2021)	Uganda	September 2020 to July 2021, with follow-up through delivery	a	b	c			Positive PCR or diagnosed probable COVID-19	Negative PCR or antibody test (recruitment)
	Crovetto (2020), Cohort I	Spain	March to May 2020, with follow-up through delivery	a	b				Positive PCR or antibody test†	Negative antibody test (ANC) and negative PCR test (delivery)
Other cohort studies	Poon (2021)	China-Hong Kong	March 2020 to January 2021	a	b	c	d	e	Positive PCR test or antibody test	Negative antibody test (ANC and delivery)
	Crovetto (2020), Cohort II	Spain	March to May 2020		b				Positive PCR or antibody test‡	Negative antibody test and PCR test (delivery)
	Bevilacqua and Laurita Longo (2020)	Italy	February 2020 to March 2021		b	c	d	e	Positive PCR test	Negative PCR test
	Nachega (2021)	Africa§ (6 countries)	March to October 2020			c		e	Positive PCR test	Negative PCR (hospital controls)
	Nunes (2021)	South Africa	April to September 2020	a¶		c	d	e	Positive PCR test	Negative PCR test
	Sakowicz (2021)	Chicago, USA	March 2020 to February 2021		b		d	e	Positive PCR test	Negative PCR test (at delivery)
	Ahlberg *et al* 19	Sweden	March to July 2020		b		d		Positive PCR test or antibody test	Negative PCR test (at delivery)
	Kalafat *et al* 22	Turkey	May to June 2020		b		d		Positive PCR test	Negative PCR test
	Brandt (2020)	New Brunswick, USA	March to June 2020		b		d		Positive PCR test	Negative PCR test

*Recruitment strategies categorised as: (a) universal screening at antenatal care, (b) universal screening at delivery, (c) hospitalised for COVID-19, (d) other COVID-19 testing for clinical concern, (e) tested based on admission for other medical reasons.

†Antibody tests were administered to women recruited at first trimester or early second trimester ANC. Participants were also administered follow-up PCR tests at labour and delivery.

‡Antibody tests were administered to all participating women at labour and delivery; most participants (85%) also received a PCR test at labour and delivery.

§Democratic Republic of Congo, Ghana, Kenya, Nigeria, South Africa, Uganda.

¶10 asymptomatic patients were tested a day during the recruitment period.

ANC, antenatal care.

**Table 2:** Description of participants contributing to the individual patient data meta-analysis

Study author (year), country	Number of COVID-19 cases/total pregnancies	Live births	Among all pregnancies		Among COVID-19 cases				
			Mean age (SD)	% obese (BMI ≥30)	Asymptomatic (%)	Onset in trimester 1 (%)	Onset in trimester 2 (%)	Onset in trimester 3 (%)	Unknown GA at onset (%)
Akelo and Tippett Barr (2021), Kenya	106/1560	805	25.62 (5.35)*	–	37.7*	1	14	51	34
Le Doare (2021), Uganda	69/532	516	25.93 (5.72)	–	4.0	1	42	54	3
Crovetto (2020), Spain, Cohort I	173/921	761	33.17 (5.3)	10.0	80.3	–	–	–	100†
Poon (2021), China-Hong Kong	25/152	155	33.00 (4.53)	–	24.0	4	28	64	4
Crovetto (2020), Spain, Cohort II	176/1304	1332	31.90 (5.78)	12.00	59.1	0†	0†	14†	86†
Bevilacqua and Laurita Longo (2020), Italy	109/2465	2413	33.74 (5.4)	15.0‡	51.4	*7*	*7*	85	0
Nachega (2021), Multi-country Africa	349/442	183	30.54 (5.72)	–	0.0	6§	18§	64§	12§
Nunes (2021), South Africa	139/781	756	30.90 (6.74)	–	12.9¶	2	22	71	5
Sakowicz (2021), USA	503/1773	1788	32.22 (5.10)	–	31.6**	5	21	73	1
Ahlberg *et al* (2020),^19^ Sweden	156/2682	2714	32.2 (5.07)	15.6††	68.6	0	3	97	0
Kalafat *et al* (2020),^22^ Turkey	77/362	346	27.15 (5.63)	–	24.7	–	–	–	100
Brandt (2020), USA	60/162	161	30.90 (6.34)	–	55.0	0	5	95	0

*This study includes 12 participants with unknown age (all COVID-negative comparisons). Two COVID-19 cases had unknown symptom status.

†Antibody testing at ANC (Cohort I) and at labour and delivery (Cohort II) was the primary method of diagnosis, thus gestational age at COVID-19 onset is unknown for almost all observations.

‡167 participants had missing BMI data (7%).

§Gestational age at COVID-19 onset was not recorded. We use trimester of hospital admission as a proxy. n=41 participants were missing trimester of hospital admission (12%).

¶Approximately 1.4% of the COVID-19 case sample in this study (Nunes, 2021, South Africa) have unknown symptom status (n=2).

**Approximately 2% of the COVID-19 case sample in this study (Sakowicz, 2021, USA) have unknown symptom status (n=8).

††100 participants had missing BMI data (4%).

ANC, antenatal care; BMI, body mass index; GA, gestational age.

### Participant characteristics

The pooled data included 1942 pregnant women with confirmed or probable SARS-CoV-2 infection during pregnancy or within 7 days of pregnancy outcome and 11 194 pregnant women who were either PCR negative at delivery (seven studies, 7274 pregnancies); antibody negative at delivery (one study, 1128 pregnancies), both antibody negative and PCR negative at delivery (one study, 127 pregnancies); antibody negative at an early ANC visit with PCR testing at delivery (one study, 748 pregnancies); negative throughout pregnancy based on repeated PCR or antibody testing offered at ANC visits and delivery (one study, 1454 pregnancies); or who were antibody and/or PCR negative at recruitment in early pregnancy with no subsequent positive test (completed for clinical concern) or clinical diagnosis of probable COVID-19 (one study, 463 pregnancies) (table 2). The total number of pregnancies included in each study ranged from 152 in China-Hong Kong (Poon, 2021) to 2682 in Sweden. [19] The mean age across all studies was 31 years, with the youngest study population in Kenya (Akelo and Tippett Barr, 2021) and the oldest study population in Italy (Bevilacqua and Laurita Longo, 2020). The prevalence of obesity ranged from 10% in Spain (Crovetto, 2020, Cohort I) to 15.6% in Sweden [19] although pre-pregnancy BMI was generally not available across studies. There were relatively few instances of SARS-CoV-2 infection identified during the first trimester; the majority of cases were identified during the third trimester (table 2). The mean age was similar between SARS-CoV-2-infected women and those in the negative comparison group (online supplemental table S3). Only four studies collected data on pre-pregnancy BMI; SARS-CoV-2-infected women were more likely to be obese (online supplemental table S3).

### Critical care indicators

Compared with pregnant women without infection, women with SARS-CoV-2 infection at any time during pregnancy had an increased risk of all outcomes related to critical care (table 3). The pooled absolute risk of ICU admission among pregnant women with SARS-COV-2 infection was 3% (95% CI 0% to 9%). Pregnant women with SARS-COV-2 infection were at a significantly increased risk of *ICU admission* (8 studies; 6660 pregnant women; RR 3.81, 95% CI 2.03 to 7.17) and *ventilation* (7 studies; 4887 pregnant women; RR 15.23, 95% CI 4.32 to 53.71). Across seven studies, about 4% of pregnant women with COVID-19 received any critical care (95% CI 0% to 13%) and they were more than five times more likely to receive critical care than their COVID-19-negative peers (7 studies; 4735 pregnant women; RR 5.48, 95% CI 2.57 to 11.72).

**Table 3:** Absolute and relative risk of adverse health outcomes comparing pregnant women with and without SARS-CoV-2 infection

Outcomes	Studies (n)	Included studies*†	Confirmed COVID-19 case		COVID-19-negative comparison		Relative risk (95% CI)¶	I^2^% (P value)
			Events/total	Pooled risk (95% CI)§	Events/total	Pooled risk (95% CI)§		
**Critical care indicators**								
ICU admission	8	c d e1* e2 f h j k	78/1299	0.03 (0.00 to 0.09)	17/5361	0.00 (0.00 to 0.01)	**3.81 (2.03 to 7.17)**	0 (0.67)
Ventilation	7	c d e1* e2 f h j	21/796	0.02 (0.01 to 0.04)	0/4091	0.00 (0.00 to 0.00)	**15.23 (4.32 to 53.71)**	0 (0.81)
Any critical care	7	c d e1* e2 f h j*	73/771	0.04 (0.00 to 0.13)	10/3964	0.00 (0.00 to 0.01)	**5.48 (2.57 to 11.72)**	0 (0.73)
Pneumonia	6	c e1* e2 f h j*	124/711	0.18 (0.05 to 0.37)	13/3862	0.01 (0.00 to 0.02)	**23.46 (3.03 to 181.39)**	91 (0)
**Maternal mortality and morbidity**								
Maternal death	10	a* c* d* e1* e2* f* g h i j*	38/368	0.07 (0.00 to 0.22)	3/1122	0.00 (0.00 to 0.02)	**7.68 (1.70 to 34.61)**	30.49 (0.24)
Haemorrhage	6	a c g h i k	109/1136	0.08 (0.04 to 0.14)	795/7273	0.07 (0.03 to 0.13)	1.22 (0.76 to 1.98)	68.35 (0.01)
Placental abruption	5	a f h j k	15/928	0.01 (0.00 to 0.03)	56/4258	0.01 (0.00 to 0.02)	1.55 (0.75 to 3.21)	0 (0.55)
Hypertensive disorders of pregnancy (diagnosed at or after COVID-19)	3	a b j	24/255	0.08 (0.01 to 0.20)	254/3396	0.06 (0.01 to 0.14)	1.33 (0.89 to 1.98)	0 (0.8)
Hypertensive disorders of pregnancy (diagnosed at any time)	10	a b c e1 e2 g h i j k	178/1570	0.09 (0.05 to 0.14)	497/9902	0.06 (0.04 to 0.09)	**1.25 (1.04 to 1.50)**	0 (0.87)
Pre-eclampsia	9	a b d e1 e2 f i j k	100/1360	0.06 (0.04 to 0.08)	324/7417	0.04 (0.02 to 0.06)	**1.42 (1.13 to 1.78)**	0 (0.62)
Eclampsia	7	a* b* e1* e2* i j* k*	0/133	–	2/613	0.00 (0.00 to 0.01)	0.92 (0.04 to 19.04)	0 (0)
Pre-eclampsia or eclampsia	10	a b c e1 e2 g h i j k	138/1570	0.07 (0.04 to 0.11)	399/9902	0.04 (0.02 to 0.05)	**1.46 (1.17 to 1.81)**	0 (0.57)
Thromboembolic disease	8	a c d* e1* e2* g* i* j*	2/265	0.01 (0.00 to 0.02)	7/4881	0.00 (0.00 to 0.00)	**5.50 (1.12 to 27.12)**	0 (0.46)
Preterm labour	6	c e1* e2 g i j	33/508	0.06 (0.03 to 0.10)	190/4674	0.04 (0.02 to 0.05)	1.59 (0.87 to 2.89)	43.18 (0.13)
Preterm labour—COVID onset <37 weeks	4	c g i j	17/223	0.07 (0.03 to 0.12)	114/3546	0.03 (0.02 to 0.04)	**2.47 (1.28 to 4.79)**	17.31 (0.3)
C-section	10	a c d e1 e2 g h i j k	499/1505	0.34 (0.28 to 0.40)	2609/9066	0.30 (0.25 to 0.36)	**1.10 (1.01 to 1.20)**	0 (0.88)
Intrapartum C-section	8	a c e1* e2 g h i j	166/792	0.22 (0.14 to 0.30)	1053/7095	0.17 (0.12 to 0.23)	1.14 (0.97 to 1.34)	0 (0.66)
**Fetal and neonatal mortality and morbidity**								
Stillbirth**	12	a b c d* e1 e2 f g h i j* k	14/1602	0.01 (0.00 to 0.02)	64/10 060	0.01 (0.00 to 0.01)	1.08 (0.53 to 2.16)	0 (0.97)
Perinatal death††	9	a c d e1 e2 f g i j*	7/931	0.00 (0.00 to 0.01)	48/8078	0.01 (0.00 to 0.01)	1.23 (0.58 to 2.61)	0 (0.93)
Early neonatal death††	9	a c d e1 e2 f g i j*	1/928	0.00 (0.00 to 0.00)	23/8071	0.00 (0.00 to 0.00)	1.37 (0.47 to 4.01)	0 (0.85)
Neonatal death††	10	a c d e1 e2 f g h i j*	4/1064	0.00 (0.00 to 0.01)	30/8118	0.00 (0.00 to 0.00)	1.71 (0.71 to 4.12)	0 (0.8)
NICU admission at birth‡‡	7	a c d e2 f g j	110/661	0.21 (0.06 to 0.41)	472/6976	0.07 (0.05 to 0.08)	**1.86 (1.12 to 3.08)**	73.78 (0)
**Adverse birth outcomes**								
Very low birth weight (<1500 g)	12	a b c d e1 e2 f g h i j* k	30/1646	0.01 (0.01 to 0.03)	169/10 129	0.01 (0.01 to 0.02)	1.12 (0.74 to 1.71)	0 (0.99)
Low birth weight (<2500 g)	12	a b c d e1 e2 f g h i j k	198/1670	0.12 (0.08 to 0.16)	926/10 260	0.09 (0.07 to 0.11)	**1.19 (1.02 to 1.40)**	0 (0.6)
Small for gestational age (3rd centile)	12	a b c d e1 e2 f g h i j k	48/1670	0.03 (0.01 to 0.05)	293/10 260	0.03 (0.02 to 0.05)	1.05 (0.77 to 1.43)	0 (0.67)
Small for gestational age (10th centile)	12	a b c d e1 e2 f g h i j k	136/1670	0.08 (0.05 to 0.12)	885/10 260	0.09 (0.07 to 0.12)	0.96 (0.80 to 1.15)	0 (0.56)
Moderate preterm birth (<34 weeks)	12	a b c d e1 e2 f g h i j k	80/1666	0.05 (0.03 to 0.07)	354/10 218	0.03 (0.02 to 0.04)	**1.37 (1.05 to 1.79)**	0 (0.84)
Moderate preterm birth (<34 weeks)—COVID onset <34 weeks‡	7	b c d g i j k	48/448	0.12 (0.04 to 0.23)	241/5623	0.04 (0.02 to 0.06)	**2.92 (1.88 to 4.54)**	34.74 (0.16)
Preterm birth (<37 weeks)	12	a b c d e1 e2 f g h i j k	234/1666	0.14 (0.10 to 0.19)	1054/10 218	0.10 (0.08 to 0.14)	**1.27 (1.07 to 1.49)**	11.96 (0.33)
Preterm birth (<37 weeks)—COVID onset <37 weeks‡	7	b c d g i j k	129/610	0.24 (0.15 to 0.35)	742/5623	0.12 (0.08 to 0.17)	**1.71 (1.28 to 2.29)**	50.31 (0.06)

*Included studies for each estimate are categorised as follows: (a) Ahlberg *et al*, Sweden^19^; (b) Akelo and Tippett Barr (2021), Kenya; (c) Bevilacqua and Laurita Longo (2020), Italy; (d) Brandt (2020), New Brunswick, USA; (e1) Crovetto (2020), Spain, Cohort I; (e2) Crovetto (2020), Spain, Cohort II; (f) Kalafat *et al*, Turkey^22^; (g) Le Doare (2021), Uganda; (h) Nachega (2021), Multi-country Africa; (i) Nunes (2021), South Africa; (j) Poon (2021), China-Hong Kong; (k) Sakowicz (2021), Chicago, USA.

†Asterisks indicate there is zero total outcome event for a given study. These studies are not included in the ‘Events/Total’ and pooled risk estimates.

‡These outcomes (preterm labour, very preterm birth before 34 weeks’ gestation and preterm birth before 37 weeks’ gestation) were included in the sensitivity analyses where we restrict confirmed COVID-19 cases to those with confirmed COVID-19 onset prior to 37 weeks’ gestation (or 34 weeks for moderate preterm birth). The full comparison group is used for each of the sensitivity analyses.

§Pooled absolute risks are calculated using Freeman-Tukey transformed proportions, pooled from all participating studies with at least one adverse event for the given outcome, using a DerSimonian-Laird random-effects inverse-variance model meta-analysis.

¶Relative risks are calculated by pooling unadjusted relative risks from all participating studies with at least one adverse event for the given outcome using a DerSimonian-Laird random-effects model meta-analysis. For any study with zero event in one arm (COVID-19 cases or COVID-negative comparisons), we used a continuity correction of 0.5.

**The outcome presented here is stillbirth occurring at or after 28 weeks’ gestational age per WHO definition.

††The outcome ‘neonatal death’ is reported by nine participating studies. However, most studies were not designed to follow-up neonates until 28 days after birth. The count of neonatal deaths is likely an underestimate.

‡‡The outcome ‘NICU Admission at Birth’ is defined as admission to the neonatal intensive care unit or the equivalent for all studies except for Crovetto (2020), Spain, Cohort II, where the outcome also includes ‘admission to high-dependency care unit’.

C-section, caesarean section; ICU, intensive care unit; NICU, neonatal intensive care unit.

### Maternal mortality and morbidity

While 10 studies collected data regarding maternal deaths, only three studies (Nachega 2021, Nunes 2021 and Le Doare 2021) recorded deaths during the study period and thus contributed information to the pooled estimate. All the remaining studies recorded zero death in both groups. Based on these three studies, women with SARS-CoV-2 infection had an increased risk of *maternal death* (10 studies; 1490 pregnant women; RR 7.68, 95% CI 1.70 to 34.61) as compared with uninfected pregnant women.

Regarding maternal morbidity, we found a greater risk for *pre-eclampsia* (9 studies; 8777 pregnant women; RR 1.42, 95% CI 1.13 to 1.78), *pre-eclampsia or eclampsia* (10 studies; 11 472 women; RR 1.46, 95% CI 1.17 to 1.81) and *thromboembolic disease* (8 studies; 5146 pregnant women; RR 5.50, 95% CI 1.12 to 27.12) among pregnant women with SARS-COV-2 infection compared with those without. We also found an increased risk for *hypertensive disorders of pregnancy* (10 studies; 11 472 pregnant women; RR 1.25, 95% CI 1.04 to 1.50) among pregnant women with SARS-CoV-2. Although most studies did not collect data on the timing of diagnosis of hypertensive disorders of pregnancy, we conducted this analysis again restricting to only those cases of hypertensive disorders of pregnancy diagnosed at or after a positive SARS-CoV-2 test; we found a similar increased risk but a wider CI (three studies representing 3651 women; RR 1.33, 95% CI 0.89 to 1.98). The risk for caesarean delivery was slightly higher among pregnant women with SARS-CoV-2 (10 studies; 10 571 pregnant women; RR 1.10, 95% CI 1.01 to 1.20). While there was no significant difference in the risk of preterm labour across both groups overall, we find an increased risk of preterm labour (<37 weeks’ gestational age) for pregnant women with SARS-CoV-2 onset before 37 weeks’ gestational age as compared with pregnant women without SARS-CoV-2 for those studies where data on gestational age at onset and preterm labour as a maternal morbidity are available (4 studies; 3769 pregnant women; RR 2.47, 95% CI 1.28 to 4.79). There was no difference between the two groups on the risk of other maternal morbidity outcomes (haemorrhage, placental abruption, eclampsia or intrapartum caesarean delivery).

### Fetal and neonatal mortality and morbidity

Among the five fetal and neonatal outcomes examined, we found an elevated risk only for NICU admission after birth among infants born to women with SARS-CoV-2 infection (7 studies; 7637 neonates; RR 1.86, 95% CI 1.12 to 3.08).

### Adverse birth outcomes

Infants born to women with confirmed or probable SARS-CoV-2 infection during pregnancy were more likely to be born *preterm* (12 studies; 11 884 live births; RR 1.27, 95% CI 1.07 to 1.49) and moderate preterm (12 studies; 11 884 live births; RR 1.37, 95% CI 1.05 to 1.79). A sensitivity analysis restricted to the seven studies recording the date of COVID-19 onset and preterm birth found a similar, although strengthened, link between SARS-CoV-2 infection and moderate preterm and preterm births. Infection during pregnancy was linked to a nearly threefold increased risk of moderate preterm birth (RR 2.92, 95% CI 1.88 to 4.54) and a near doubling of the risk in preterm birth (RR 1.71, 95% CI 1.28 to 2.29) (table 3). Infants born to women with SARS-CoV-2 infection during pregnancy were also more likely to be low birth weight (<2500 g) (12 studies; 11 930 neonates; RR 1.19, 95% CI 1.02 to 1.40).

### Secondary analysis (symptomatic COVID-19 cases)

We conducted a secondary analysis restricted to only symptomatic infections as compared with SARS-CoV-2 uninfected pregnant women; asymptomatic infections were excluded from this analysis. Similar to the primary analysis, we found that pregnant women with symptomatic infections were more likely than uninfected pregnant women to be admitted to the ICU, require ventilation or receive critical care. The risk of maternal death was also significantly higher. They were also more likely to be diagnosed with pneumonia, hypertensive disorders of pregnancy, pre-eclampsia, pre-eclampsia or eclampsia, or thromboembolic disease. They were more likely to experience preterm labour and to have a caesarean delivery or require an intrapartum caesarean delivery. Infants born to women with symptomatic SARS-CoV-2 during pregnancy were more likely to be born very low birth weight, low birth weight, moderate preterm and preterm; they were also more likely to be admitted to the NICU as compared with infants born to women without COVID-19 during pregnancy (table 4).

**Table 4:** Relative risk of outcomes comparing COVID-19 cases (symptomatic cases only) versus COVID-negative pregnancies

Outcome	Studies (n)	Included studies*†	Symptomatic RR (95% CI)
ICU admission	8	c d e1* e2 f h j k	**4.88 (2.57 to 9.27)**
Ventilation	7	c d e1* e2 f h j	**24.09 (6.85 to 84.77)**
Critical care	7	c d e1* e2 f h j*	**8.47 (3.37 to 21.28)**
Pneumonia	6	c e1* e2 f h j*	**34.58 (3.36 to 356.13)**
Maternal death	10	a* c* d* e1* e2* f* g h i j*	**8.48 (1.70 to 42.21)**
Haemorrhage	6	a c g h i k	1.30 (0.81 to 2.10)
Placental abruption	5	a f h j* k	2.08 (0.95 to 4.53)
Hypertensive disorders of pregnancy (diagnosed at or after COVID-19)		a b j	**1.74 (1.01 to 3.00)**
Hypertensive disorders of pregnancy (diagnosed at any time)	10	a b c e1 e2 g h i j k	**1.28 (1.03 to 1.59)**
Pre-eclampsia	9	a b d e1 e2 f i j k	**1.58 (1.20 to 2.08)**
Eclampsia	7	a* b* e1* e2* i j* k*	1.07 (0.05 to 22.17)
Pre-eclampsia or eclampsia	10	a b c e1 e2 g h i j k	**1.63 (1.26 to 2.11)**
Thromboembolic disease	8	a c d* e1* e2* g* i* j*	**9.64 (1.69 to 54.97)**
Preterm labour	6	c e1* e2 g i j	**1.87 (1.06 to 3.32)**
Preterm labour (COVID-19 onset <37 weeks)	4	c g i j	**2.71 (1.25 to 5.85)**
Caesarean section	10	a c d e1 e2 g h i j k	**1.16 (1.04 to 1.29)**
Intrapartum C-section	8	a c e1* e2 g h i j	**1.27 (1.06 to 1.52)**
Stillbirth	12	a b c d* e1 e2 f g h i j* k	1.35 (0.62 to 2.96)
Perinatal death	9	a c d e1 e2 f g i j*	1.45 (0.62 to 3.43)
Early neonatal death	9	a c d e1 e2* f g i j*	1.89 (0.61 to 5.9)
Neonatal death	10	a c d e1 e2* f g h i j*	1.93 (0.71 to 5.25)
NICU admission at birth	7	a c d e2 f g j	**2.12 (1.31 to 3.43)**
Very low birth weight (<1500 g)	12	a b c d e1 e2 f g h i j* k	**1.67 (1.07 to 2.62)**
Low birth weight (<2500 g)	12	a b c d e1 e2 f g h i j k	**1.32 (1.09 to 1.59)**
Small for gestational age (3rd)	12	a b c d e1 e2 f g h i j k	1.22 (0.86 to 1.71)
Small for gestational age (10th)	12	a b c d e1 e2 f g h i j k	1.05 (0.85 to 1.30)
Moderate preterm birth (<34 weeks)	12	a b c d e1 e2 f g h i j k	**1.62 (1.20 to 2.17)**
Moderate preterm birth (<34 weeks) (COVID-19 onset <34 weeks)‡	7	b c d g i j k	**3.12 (1.94 to 5.02)**
Preterm birth (<37 weeks)	12	a b c d e1 e2 f g h i j k	**1.41 (1.15 to 1.73)**
Preterm birth (<37 weeks) (COVID-19 onset <37 weeks)‡	7	b c d g i j k	**1.70 (1.22 to 2.36)**

*Included studies for each estimate are categorised as follows: (a) Ahlberg *et al*, Sweden^19^; (b) Akelo and Tippett Barr (2021), Kenya; (c) Bevilacqua and Laurita Longo (2020), Italy; (d) Brandt (2020), New Brunswick, USA; (e1) Crovetto (2020), Spain, Cohort I; (e2) Crovetto (2020), Spain, Cohort II; (f) Kalafat *et al*, Turkey^22^; (g) Le Doare (2021), Uganda; (h) Nachega (2021), Multi-country Africa; (i) Nunes (2021), South Africa; (j) Poon (2021), China-Hong Kong; (k) Sakowicz (2021), Chicago, USA.

†Asterisks indicate there is zero total event for a given study. These studies are not included in the ‘Events/Total’ and pooled risk estimates.

‡These outcomes (preterm labour, moderate preterm birth before 34 weeks’ gestation and preterm birth before 37 weeks’ gestation) were included in the sensitivity analyses where we restrict COVID-19 cases to those with confirmed onset prior to 37 weeks’ gestation (or 34 weeks for moderate preterm birth). The full comparison group is used for each of the sensitivity analyses.

C-section, caesarean section; ICU, intensive care unit; NICU, neonatal intensive care unit; RR, relative risk.

Additional sensitivity analyses comparing the results of this meta-analysis to results of eligible studies in the PregCOV-19 Living Systematic Review and comparing pooled estimates among PMA studies using different definitions of stillbirth are presented in the online supplemental tables S4 and S5, respectively.

We found the majority of included studies and outcomes to be at low risk of bias (table 5). Three studies received a star for all domains for all outcomes, indicating the lowest risk of bias; the majority of other studies had only one domain where a higher risk of bias was a concern. The most common reason a study was considered at higher risk of bias was related to selection of the exposed group (SARS-CoV-2 infection); in seven studies, more than half of the SARS-CoV-2-infected women were identified in a way that was potentially not representative of the general pregnant population in the community, such as testing based on recent travel or clinical concern, or clinical diagnosis of probable COVID-19 based on symptoms (online supplemental table S6). Three studies were deemed at higher risk of bias because more than 10% of women had incomplete information about the pregnancy outcome, and three studies were deemed at higher risk of bias because more than 10% of participants were missing a particular outcome (online supplemental tables S7 and S8).

**Table 5:** Summary of risk of bias assessment for individual studies based on an adapted Newcastle-Ottawa Scale

	Exposure*				Outcome†				Total stars
	Representativeness of exposed cohort	Selection of non-exposed cohort	Ascertainment of exposure (SARS-CoV-2 infection)	Ascertainment of control (SARS-CoV-2 negative)	Outcome assessment data source	Adequacy pregnancy follow-up		Data completeness‡	
Akelo and Tippett Barr (2021)	*	*	*	*	*	§	(a) Critical care	N/A	N/A
							(b) Maternal mortality and morbidity	*	6/7
							(c) Fetal and neonatal mortality	N/A	N/A
							(d) Adverse birth outcomes	¶	5/7
Le Doare (2021)	**	*	††	*	*	*	(a) Critical care	N/A	N/A
							(b) Maternal mortality and morbidity	*	5/7
							(c) Fetal and neonatal mortality	*	5/7
							(d) Adverse birth outcomes	*	5/7
Crovetto (2020), Cohort I	*	*	*	*	*	§	(a) Critical care	*	6/6
							(b) Maternal mortality and morbidity	*	6/7
							(c) Fetal and neonatal mortality	*	6/7
							(d) Adverse birth outcomes	*	6/7
Poon (2021)	**	*	*	*	*	*	(a) Critical care	*	5/6
							(b) Maternal mortality and morbidity	*	6/7
							(c) Fetal and neonatal mortality	*	6/7
							(d) Adverse birth outcomes	*	6/7
Crovetto (2020), Cohort II	*	*	*	*	*	*	(a) Critical care	*	6/6
							(b) Maternal mortality and morbidity	*	7/7
							(c) Fetal and neonatal mortality	*	7/7
							(d) Adverse birth outcomes	*	7/7
Bevilacqua and Laurita Longo (2020)	**	*	*	*	*	*	(a) Critical care	*	5/6
							(b) Maternal mortality and morbidity	*	6/7
							(c) Fetal and neonatal mortality	*	6/7
							(d) Adverse birth outcomes	*	6/7
Nachega (2021)	**	*	*	*	*	§	(a) Critical care	¶	4/6
							(b) Maternal mortality and morbidity	¶	4/7
							(c) Fetal and neonatal mortality	*	5/7
							(d) Adverse birth outcomes	¶	4/7
Nunes (2021)	**	*	*	*	*	*	(a) Critical care	N/A	N/A
							(b) Maternal mortality and morbidity	*	6/7
							(c) Fetal and neonatal mortality	*	6/7
							(d) Adverse birth outcomes	*	6/7
Sakowicz *et al* 23	**	*	*	*	*	*	(a) Critical care	*	5/6
							(b) Maternal mortality and morbidity	*	6/7
							(c) Fetal and neonatal mortality	¶	5/7
							(d) Adverse birth outcomes	*	6/7
Ahlberg *et al* 19	*	*	*	*	*	*	(a) Critical care	N/A	N/A
							(b) Maternal mortality and morbidity	*	7/7
							(c) Fetal and neonatal mortality	*	7/7
							(d) Adverse birth outcomes	*	7/7
Kalafat *et al* 22	**	*	*	*	*	*	(a) Critical care	*	5/6
							(b) Maternal mortality and morbidity	*	6/7
							(c) Fetal and neonatal mortality	*	6/7
							(d) Adverse birth outcomes	*	6/7
Brandt (2020)	*	*	*	*	*	*	(a) Critical care	*	6/6
							(b) Maternal mortality and morbidity	*	7/7
							(c) Fetal and neonatal mortality	*	7/7
							(d) Adverse birth outcomes	*	7/7

Stars (*) indicate a study is at lower risk of bias in a given domain.

*See online supplemental table S6 for detailed risk of bias assessment related to selection of the exposed and unexposed cohorts for individual studies.

†See online supplemental table S6 for detailed risk of bias assessment related to outcome assessment for individual studies.

‡See online supplemental table S8 for a description of follow-up by study and review of missing data by outcome.

§Pregnancy follow-up domain deemed at higher risk of bias because <90% of pregnancy outcomes had been ascertained at the time of data transfer.

¶Data completeness domain deemed at higher risk of bias because one or more outcomes in this category had missing data for 11%–25% of participants.

**Representativeness of the exposed cohort domain deemed at higher risk of bias because 50% or more of the cases were identified using a method that was only somewhat representative of all SARS-CoV-2-infected pregnant women in the community (eg, pregnant women tested at antenatal care of delivery based on symptoms or travel; pregnant women tested for antibodies during routine screening; medical records of pregnant women hospitalised for any reason, excluding delivery).

††Ascertainment of exposure (SARS-CoV-2 infection) domain deemed at higher risk of bias because a proportion of COVID-19-positive cases were identified through clinical diagnosis or radiography consistent with WHO case definitions of probable and suspected cases.

N/A, not available.

## Discussion

Our IPD meta-analysis confirms findings from a growing body of published literature that SARS-CoV-2 infection during pregnancy increases the risk of maternal death and imparts an increased risk for adverse health outcomes for both pregnant women and their fetuses and neonates.

Compared with a contemporaneous group of pregnant women who tested negative for SARS-COV-2 infection, those with infection at any time during pregnancy had a higher risk for all critical care indicators, maternal mortality and several morbidity outcomes such as hypertensive disorders of pregnancy, pre-eclampsia or eclampsia, preterm labour and thromboembolic disease. Our findings are consistent with a living systematic review that included studies using concurrent or historical controls which found that women with COVID-19 during pregnancy had an increased risk of ICU admission and all-cause mortality.^1^ A recent multinational cohort study (the INTERCOVID study) including data from 706 SARS-CoV-2-infected pregnancies and 1424 pregnancies without a known diagnosis from 43 institutions in 18 countries found similar increased risks of ICU admission and all-cause mortality linked with COVID-19 during pregnancy. The INTERCOVID study additionally found women with COVID-19 were at higher risk for pre-eclampsia or eclampsia and severe infections (RR 3.38; 95% CI 1.63 to 7.01).^4^ Other studies have also reported that COVID-19 is linked with pre-eclampsia or eclampsia.^4 5 26^

There is widespread disagreement about the biological plausibility that SARS-CoV-2 infection can induce hypertensive disorders of pregnancy, including pre-eclampsia. Some have hypothesised that altered ACE2 expression linked to COVID-19, or the systemic inflammation and hypercoagulable state common in COVID-19, may increase the risk of pre-eclampsia.^27^ While others have suggested that SARS-CoV-2 infection may lead to a pre-eclampsia-like syndrome that will resolve along with the infection (rather than delivery),^28^ clinicians do not commonly measure angiogenic factors such as the soluble fms-like tyrosine kinase-1/placental growth factor that can differentiate between true pre-eclampsia and pre-eclampsia-like symptoms.^29 30^ Others have suggested the link between COVID-19 and hypertensive disorders of pregnancy is driven by screening bias.^31^ In general, people who face increased risks of SARS-CoV-2 infection are also at higher risk for other comorbidities such as hypertension, obesity, diabetes and pregnancy complications such as pre-eclampsia. Hence, associations between infection and adverse outcomes may be the result of residual confounding. We attempted to address whether people with hypertensive disorders of pregnancy were more likely to be screened, and thus test positive, through our sensitivity analysis including only diagnoses that occurred at or after the SARS-CoV-2 test positive date; the effect estimate was similar to primary analysis, although the CI was much wider given that only three studies contributed data to the sensitivity analysis. Determining whether a true causal link exists and elucidating the potential pathophysiology of hypertensive disorders of pregnancy among women with COVID-19 is needed to strengthen patient care and management. However, the higher risks reported here are similar to those reported by other studies^4 5 26^ and are consistent with the practice of prompt, precautionary monitoring of hypertensive women with SARS-CoV-2 infection.

Our analysis also revealed that neonates born to women with a SARS-CoV-2 infection had a significantly higher risk for a moderately preterm (<34 weeks) or preterm (<37 weeks) birth, though we did not distinguish between spontaneous and iatrogenic preterm births. These findings are consistent with other studies. Based on 18 studies in the living systematic review, COVID-19 during pregnancy is linked with a 47% increased risk of preterm birth; SARS-CoV-2 infected women in the INTERCOVID study had a similar increased risk of preterm birth and 97% increased risk of having a medically indicated preterm birth.^1 4^ Notably, we did not find any link between SARS-CoV-2 infection during pregnancy and being born small for gestational age. The INTERCOVID study, one of the few published studies to examine a similar suite of outcomes, has similar findings.^4^ Taken together, these findings suggest no association between SARS-CoV-2 infection during pregnancy and intrauterine growth restriction, although the question should be examined in more detail considering the timing and severity of infection during pregnancy.

We did not find a link between SARS-CoV-2 infection during pregnancy and an increased *risk of stillbirth* at or beyond 28 weeks’ gestation, based on analysis of 78 cases of stillbirth (14 in the COVID-19 group). This is in contrast with the living systematic review that reported that women with COVID-19 had 2.84 times the risk of stillbirth as compared with their uninfected peers, although this was based on only 35 stillbirths (nine in the COVID-19 group).^1^ A national study of more than 340 000 pregnancies in England also found a higher risk of stillbirth (adjusted OR 2.17, 95% CI 1.96 to 2.42).^5^ These inconsistent findings may be partly due to analytical choices. For example, we defined stillbirth as fetal death at or beyond 28 weeks’ gestational age^32^ while other studies used an earlier cut-off; even so, we did not find a significant difference within the PMA studies using different definitions of stillbirth (online supplemental table S5). We also excluded studies with historical controls from our analysis, and we did not use a continuity correction for zero total event study in our meta-analysis because this can cause bias when the exposed and unexposed groups are not equal in size.^33^ The design of included studies may also influence our findings. A study in Sweden compared estimates for facilities that had universal screening at ANC or delivery versus those obtained from facilities with non-universal testing policies; they found no link between SARS-CoV-2 infection and stillbirth in the universal screening analysis, but a strong relationship between infection and increased risk of stillbirth in the non-universal screening analysis.^6^ Finally, a recent report by the US Centers for Disease Control and Prevention suggests that the Delta variant is associated with a higher risk for stillbirth than earlier SARS-CoV-2 variants.^34^ Given stillbirth is a rare outcome, additional data are needed to understand the potential risk and whether risk varies based on the timing and severity of SARS-CoV-2 infection.

Our study is intended to provide robust and high-quality estimates of the impacts of SARS-CoV-2 infection during pregnancy as compared with uninfected pregnancies. The IPD meta-analysis includes both unpublished and previously published data that were uniformly processed and analysed using a harmonised set of outcomes. We also included an expanded set of maternal morbidity outcomes that have not been extensively studied. The unpublished data include information from five countries in sub-Saharan Africa; no data from sub-Saharan Africa were previously available for inclusion in the current living systematic review.^1^ Further, the IPD meta-analysis includes newer data (through July 2021) and some study designs at lower risk of potential bias. For example, the data from Akelo and Tippett Barr in Kenya, Le Doare in Uganda and Crovetto Cohort I study in Spain include data from prospective pregnancy cohorts with repeated testing throughout pregnancy. The data from Poon in China-Hong Kong and the Crovetto Cohort II study in Spain include a large control group that is antibody negative throughout pregnancy. Together, these studies provide a large comparison group that likely never had a SARS-CoV-2 infection during pregnancy. In the remaining studies, the comparison group includes pregnancies that were confirmed PCR negative at a single time point. These studies nonetheless offer an improvement over others that use a comparison group defined by the absence of a positive test, rather than a confirmed negative test. Several newer studies also included study sites with universal screening at ANC or delivery which makes these cohorts better representative of the general pregnant population; they identify cases at all gestational ages and address some concerns regarding bias that is introduced when only symptomatic women or those with severe morbidities are more likely to receive a test.

Our study is not without limitations. The possibility of selection bias remains, given that selection of pregnant women with a COVID-19 diagnosis depended on when and how the participants were tested for SARS-CoV-2; this changed over time across sites along with the availability of test kits. However, our risk of bias assessment carefully documents the methods for recruiting exposed and unexposed study participants and suggests that most participants across most studies were sampled in a representative way. Further, this analysis does not consider the differential impact of SARS-CoV-2 variants that have emerged since the onset of the pandemic because sequencing data was not available for individual patients in this study. Additionally, the majority of studies included in this analysis conducted recruitment only during a time period where a single variant was dominant at the national level (online supplemental figure S1). Another serious concern is related to incomplete follow-up for some outcomes such as maternal mortality through 42 days post partum and neonatal mortality through 28 days following birth. Most of the studies had partial follow-up, likely causing undercounting of events. Another potential limitation is the use of site-specific definitions of critical care indicators, which might introduce misclassification bias. However, it is reassuring that our findings regarding critical care indicators are not substantively different from our findings regarding maternal, fetal and neonatal mortality, which were defined using WHO criteria.

## Conclusion

Taken together, this analysis of 12 studies including 13 136 pregnant women from 12 countries indicates that SARS-CoV-2 infection at any time during pregnancy increases the risk of maternal mortality, severe maternal morbidities and adverse newborn outcomes. These findings underscore the need for global efforts to prevent COVID-19 during pregnancy through targeted administration of vaccines and non-pharmaceutical interventions. Further efforts are needed to advance our understanding of the best clinical care and management strategies for SARS-CoV-2-infected pregnant women and their newborns. As more data become available, we will update these findings as per the published protocol.

## Data Availability

No data are available. Individual patient data should be requested from the original or parent study investigators.
