# Supplementary material for: Adverse maternal, fetal, and newborn outcomes among pregnant women with SARS-CoV-2 infection: an individual participant data meta-analysis
Source: BMJ Glob Health. 2023 Jan 3;8(1):e009495. doi: 10.1136/bmjgh-2022-009495 (PMC9895919; doi:10.1136/bmjgh-2022-009495)
Supplement: Supplementary data [file bmjgh-2022-009495supp001.pdf]

Supplementary Material

Table of Contents

Table S1. Description of differences between prior publications and data contributed to the PMA
Table S2. Description of the adapted Newcastle Ottawa scale for assessing risk of bias
Figure S1: Timeline of recruitment by study compared to dominant SARS-CoV-2 variant data
Table S3. Comparison of selected demographic characteristics of pregnant women with and without Covid-19 diagnosis
Table S4. Sensitivity analysis comparing pooled PMA results to pooled results of eligible studies not recruited from the PregCOV-19 Living Systematic Review
Table S5. Sensitivity analysis comparing pooled absolute and relative risk of adverse outcomes using multiple definitions of stillbirth
Table S6. Detailed risk of bias assessment related to selection of the exposed and unexposed cohorts for individual studies
Table S7. Detailed risk of bias assessment related to outcome assessment for individual studies
Table S8. Description of Follow-up by Study and Review of Missing Data by Outcome

Table S1: Description of differences between prior publications and data contributed to the PMA

PMA Data Contributor	*Description of Re-analysis of Published Data*	*Citation*
Ahlberg et al, 2020 (Sweden)	The data submitted to the PMA by Ahlberg et al are published in the research letter by Ahlberg et al (2020).<br><br>For this analysis, we collaborated with investigators to re-analyze the data submitted according to the PMA protocol. The original analysis conducted in this study focused on a smaller sample size (n= 759) after the investigators used propensity score matching to match COVID-negative controls with COVID-positive cases. For the PMA, we use the full sample of patients presenting at labor and delivery at Karolinska University Hospital between March 25 and July 24 2020 (n=2,682).	Ahlberg, M, Neovius, M, Saltvedt, S, Söderling, J, Pettersson, K, Brandkvist, C, Stephansson, O. Association of SARS-CoV-2 Test Stattus and Pregnancy Outcomes. *JAMA*. 2020 Sept; 324(17): 1782-1785. Available from: doi: 10.1001/jama.2021.5775
Bevilacqua et al, 2020 (Italy)	The data submitted to the PMA by Bevilacqua et al are also published as a subset of the data in the PregOuTCOV Study, an international multicenter retrospective cohort in Europe (Badr et al, 2021).<br><br>For this analysis, we re-analyze the data submitted by Bevilacqua et al according to the PMA protocol. Further, this analysis excludes 728 observations collected in the original study that were asymptomatic and *not* tested for COVID-19, resulting in a sample size of n=2465 for this study.	Badr, DA, Picone, O, Bevilacqua, E, Carlin, A, Meli, F, Sibiude, J, et al. Severe Acute Respiratory Syndrome Coronavisu 2 and Pregnancy Outcomes According to Gestational Age at Time of Infection. *Emerging Infectious Diseases*. 2021 Oct; 27(10): 2535-2543. Available from: https://dx.doi.org/10.3201%2Feid2710.211394
Brandt, 2020 (USA - New Brunswick)	The data submitted to the PMA by Brandt are published in the study Brandt et al (2021).<br><br>For this analysis, we re-analyze the data submitted according to the PMA protocol. Further, this analysis excludes 21 observations collected in the original study including: 1 COVID-positive observation where COVID-19 onset was more than 1 week after the pregnancy outcome; 20 COVID-negative observations that were assumed negative for COVID-19 but did not receive a PCR test due to recruitment for the study early in the pandemic before testing was widely available (March - early April 2020). This results in a sample size of n=162 for this contributing study in the PMA.	Brandt, JS, Hill, J, Reddy, A, Schuster, M, Patrick, HS, Rosen, T, et al. Epidemiology of coronavirus disease 2019 in pregnancy: risk factors and associations with adverse maternal and neonatal outcomes. *American Journal of Obstetric Gynecology*. 2021 Apr; 224(4): 389.e1-389.e9. Available from: doi: 10.1016/j.ajog.2020.09.043
Crovetto et al, 2020 (Spain)	The data submitted to the PMA by Crovetto et al are published in the study Crovetto et al (2020).<br><br>For this analysis, we re-analyze the data submitted according to the PMA protocol. Further, for this analysis, Crovetto et al identified 32 additional COVID-positive observations among those who were initially identified as COVID-negative at early pregnancy screening and then tested positive during follow-up PCR testing at labor and delivery (Crovetto et al follow-up study forthcoming). For the PMA, we consider the Crovetto et al study as two separate cohort studies based on the distinct study designs for each cohort, with a sample size of n=921 for Cohort I and n=1,304 for Cohort II.	Crovetto, F, Crispi, F, Llurba, E, Pascal, R, Larroya, M, Trilla, C, et al. Impact of SARS-CoV-2 infection on pregnancy outcomes: A population-based study. *Clinical Infectious Diseases*. 2021 Feb; 73(10): 1768-1775. Available from: https://doi.org/10.1093/cid/ciab104
Kalafat 2020 (Turkey)	The data submitted to the PMA by Kalafat are also published as a subset of the data in the previously published study Kalafat, Yassa, Koc, Tug, and the TULIP Collaboration, 2020.<br><br>For this analysis, we re-analyze the data submitted by Kalafat according to the PMA protocol, with a sample size n=362 (collected in Istanbul, Turkey).	Kalafat, E, Yassa, M, Koc, A, Tug, N, the TULIP collaboration. Utility of lung ultrasound assessment for probable SARS-CoV-2 infection during pregnancy and universal screening of asymptomatic individuals. *Ultrasound in Obstetrics & Gynecology*. 2020 Sept; 56(4): 624-626. Available from: https://doi.org/10.1002/uog.23099
Sakowicz et al, 2021 (USA – Chicago)	A subset of the data submitted to the PMA by Sakowicz et al are published in the study Sakowicz et al, 2020. The overlapping data include all COVID-negative pregnancies submitted to the PMA (n=1,270) and all COVID-positive pregnancies delivered prior to May 31, 2020 (n=101). Sakowicz et al also submitted additional COVID-positive cases to the PMA delivered on or after June 1, 2020 (n=402) that are not included in this original publication.<br><br>For this analysis, we re-analyzed the data according to the PMA protocol, retaining all observations submitted by Sakowicz et al (n=1773).	Sakowicz, A, Ayala, AE, Ukeje, CC, Witting, CS, Grobman, WA, Miller, ES. Risk factors for severe acute respiratory syndrome coronavirus 2 infection in pregnant women. *American Journal of Obstetrics and Gynecology MFM*. 2020 Nov; 2(4): 100198. Available from: doi: 10.1016/j.ajogmf.2020.100198

Table S2: Description of the adapted Newcastle Ottawa Scale for assessing risk of bias

	Participant Selection				Outcome Assessment	
	**Representativeness of the exposed cohort (Confirmed COVID-19 cases)**	**Selection of the non-exposed cohort (COVID-negative comparisons)**	**Ascertainment of exposure (COVID-19 Cases)**	**Ascertainment of control (COVID-negative comparison group)**	**Adequacy of follow up for pregnancy outcome**	**Adequacy of data completeness (by outcome group)**
**Lower risk of bias**<br><br>(earns a quality star in the Newcastle Ottawa Scale)	a) At least 50% of the cases were identified using a method truly representative of the COVID-exposed (confirmed/suspected) pregnant women in the community (e.g., pregnant women universally tested when presenting for delivery at the hospital; pregnant women universally tested during antenatal care visits as part of routine screening)	a) Drawn from the same community and same time (e.g., calendar time or gestational age) as the exposed cohort	a) Viral test indicating active infection (e.g., PCR test, antigen test)<br><br>b) Serology/antibody test with confirmed onset during pregnancy based on date of pandemic and gestational age	a) Viral test indicating the absence of an active infection at a single or multiple times points (e.g., PCR test, antigen test)<br><br>b) Serology/antibody test indicating no infection during pregnancy	a) >90% of pregnancy outcomes ascertained	a) Complete follow up: data available for ≥99% of participants<br><br>b) Subjects lost to follow up unlikely to introduce bias (<10% of participants with missing data)
**Higher risk of bias**	b) 50% or more of the cases were identified using a method a somewhat representative of the COVID-19-infected pregnant persons in the community (e.g., pregnant women tested at antenatal care of delivery based on symptoms or travel; pregnant women tested for antibodies during routine screening; medical records of pregnant women hospitalized for any reason, excluding delivery).<br><br>c) selected group of pregnancies (e.g., nurses, volunteers)	b) Drawn from a different sampling frame or time (e.g., calendar time or gestational age)	c) Clinical diagnosis or radiography consistent with WHO case definitions of probably and suspected cases<br><br>d) Self report	c) Absence of a positive test (subject is excluded from this analysis)	b) 75-90% of pregnancy outcomes ascertained<br><br>c) <75% of pregnancy outcomes ascertained	c) Data missing for 11-25% of participants<br><br>d) > 25% of participants are missing data (outcome is excluded from this analysis)

Figure S1. Timeline of recruitment by study compared to dominant SARS-CoV-2 variant data [1,2,3]

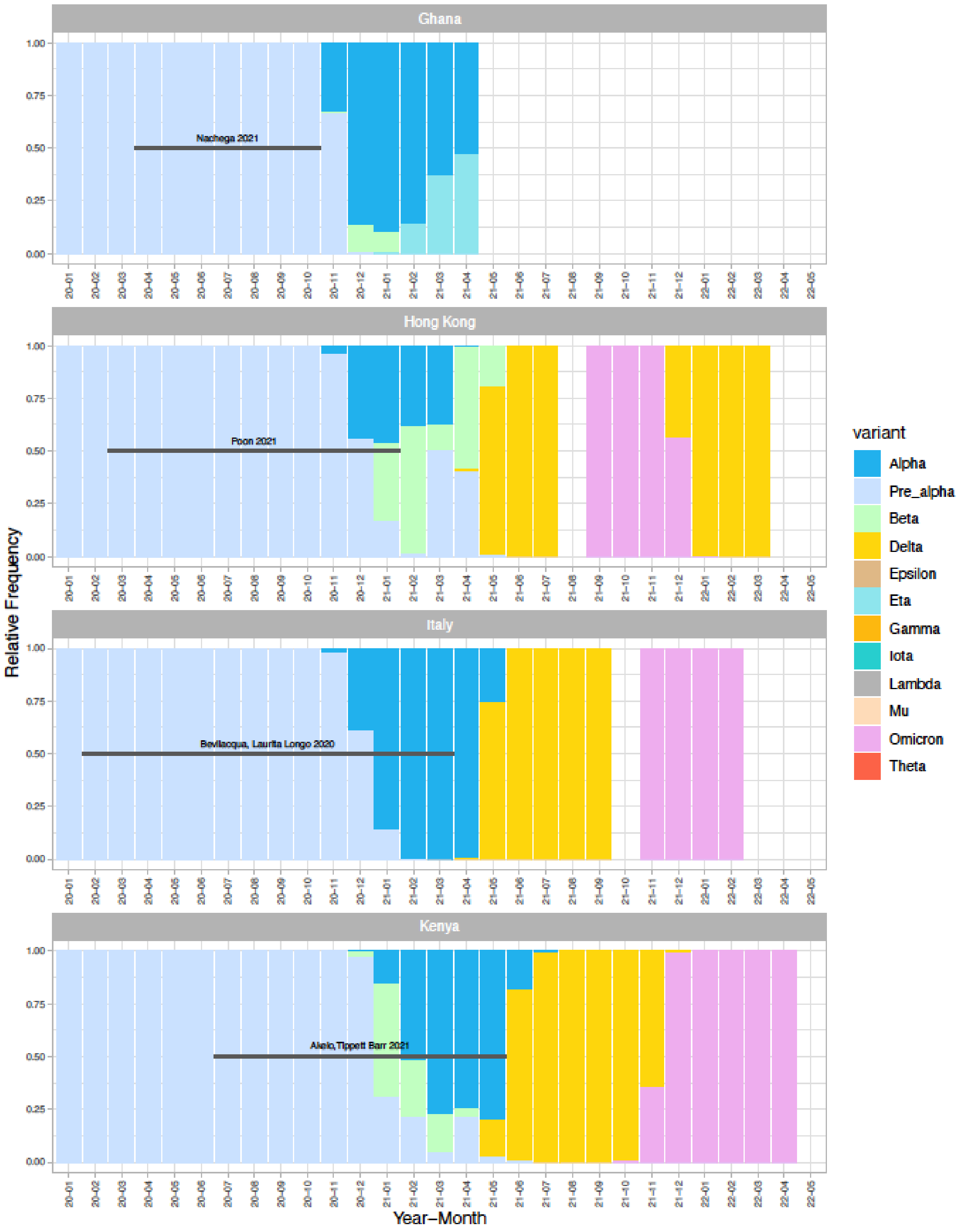

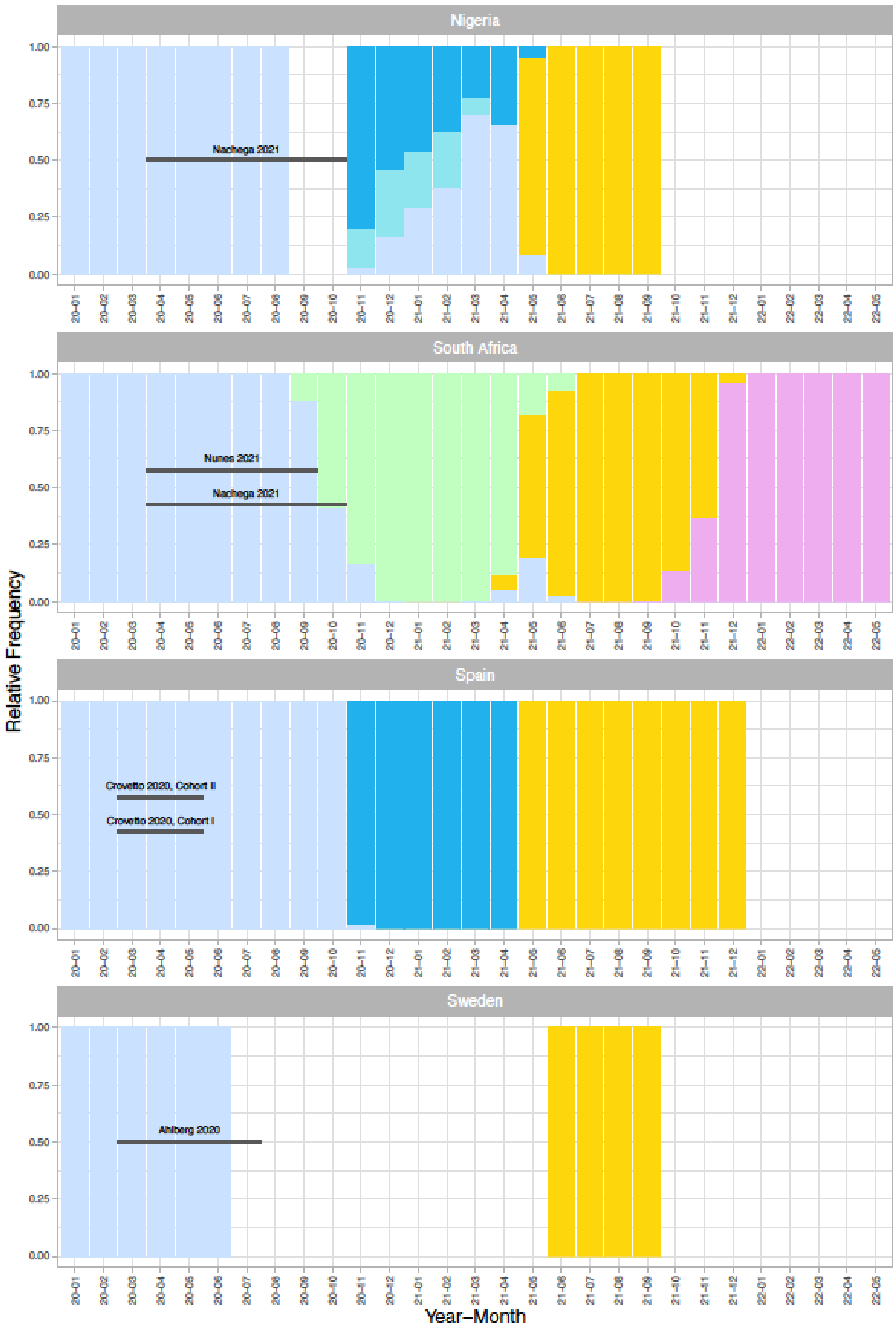
Nigeria
Nachega 2021
South Africa
Nunes 2021
Nachega 2021
Spain
Crovetto 2020, Cohort II
Crovetto 2020, Cohort I
Sweden
Ahlberg 2020
Relative Frequency
Year–Month

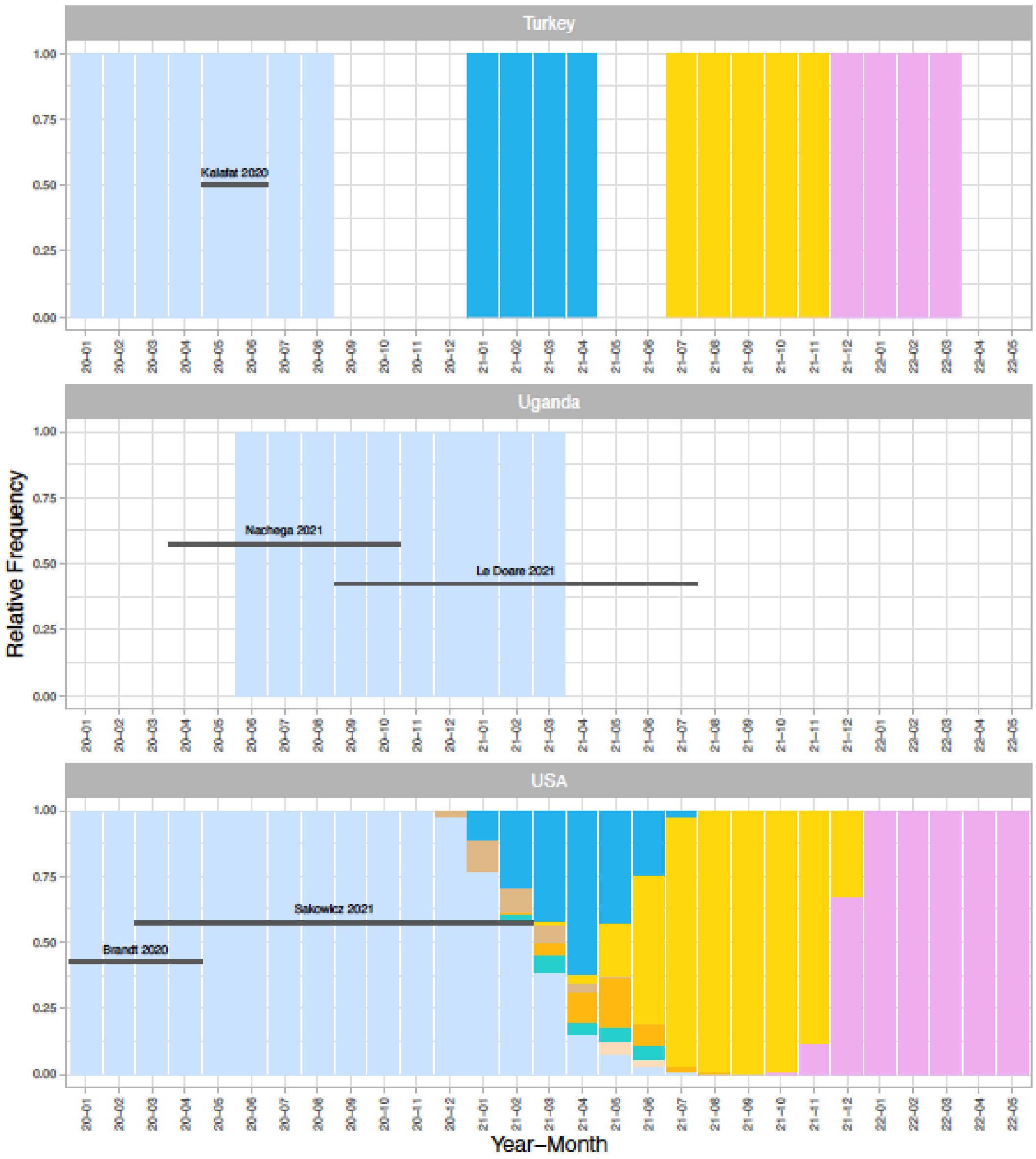

[1] Variant profile over the months for each country was generated using publicly available data from Nextstrain.org
[2] Democratic Republic of Congo variant data not available publicly
[3] White space on individual facets indicate lack of variant data by month availability at Nextstrain.org

Table S3: Comparison of selected demographic characteristics of pregnant women with and without Covid-19 diagnosis

	Mean age (SD)		% Obese	
**Study Author Year, Country**	COVID-19 Cases	COVID-19 Negative Comparison	COVID-19 Cases	COVID-19 Negative Comparison
Akelo, Tippett Barr 2021, Kenya	26.5 (5.2)	25.6 (5.4) [1]	--	--
Le Doare, 2021, Uganda	27.3 (5.8)	25.7 (5.7)	--	--
Crovetto 2020, Spain - Cohort I	32.7 (5.4)	33.3 (5.3)	11.6%	9.1%
Poon 2021, China-Hong Kong	33.7 (5.4)	32.9 (4.4)	--	--
Crovetto 2020, Spain - Cohort II	32.0 (6.2)	31.9 (5.7)	13.6%	11.2%
Bevilacqua, Laurita Longo 2020, Italy	32.6 (5.7)	33.8 (5.4)	19.3%	15.2%
Nachega 2021, Afrehealth	30.7 (5.8) [2]	30.1 (5.5)	--	--
Nunes 2021, South Africa	31.8 (6.6)	30.7 (6.8)	--	--
Sakowicz, 2021, USA	30.8 (5.8)	32.8 (4.7)	--	--
Ahlberg, 2020, Sweden [3]	32.1 (4.8)	32.3 (5.1)	25.0%	15.0%
Kalafat 2020, Turkey	28.0 (5.9)	26.9 (5.5)	--	--
Brandt 2020, USA	30.3 (6.5)	31.2 (6.3)	--	--

[1] n=12 observations missing age data
[2] n=2 observations missing age data
[3] n=100 observations had missing data on BMI (across the two groups)

Table S4. Sensitivity analysis comparing pooled PMA results to pooled results of eligible studies not recruited from the PregCOV-19 Living Systematic Review

	PMA Studies			PregCOV-19 Studies[1]			Overall Results		
**Outcomes**	**N Studies**	**Relative Risk[2] (95% CI)**	**I^2% (p value)**	**N Studies**	**Relative Risk[2] (95% CI)**	**I^2% (p value)**	**N Studies**	**Relative Risk[2] (95% CI)**	**I^2% (p value)**
ICU admission	8	**3.81 (2.03, 7.17)**	0 (0.67)	19	**4.56 (3.04, 6.84)**	57.7 (0.001)	27	**4.47 (3.16, 6.32)**	48.6 (0.004)
All-cause mortality	10	**7.68 (1.70, 34.61)**	30.5 (0.24)	18	**5.57 (1.46, 21.22)**	81.7 (0)	28	**6.53 (2.42, 17.62)**	72.4 (0.001)
C-section	10	**1.10 (1.01, 1.20)**	0 (0.88)	40	1.03 (0.96, 1.10)	40.1 (0.005)	50	1.05 (0.99, 1.10)	30.6 (0.02)
Stillbirth [3]	12	1.08 (0.53, 2.16)	0 (0.97)	18	**1.66 (1.26, 2.20)**	0 (0.73)	30	**1.57 (1.21, 2.03)**	0 (0.93)
Neonatal death [4]	10	1.71 (0.71, 4.12)	0 (0.8)	17	1.95 (0.89, 4.29)	0 (0.83)	27	**1.84 (1.02, 3.31)**	0 (0.81)
Admission to neonatal unit at birth [5]	7	**1.86 (1.12, 3.08)**	73.8 (0)	22	**1.57 (1.12, 2.20)**	83.3 (0)	29	**1.64 (1.24, 2.17)**	81.2 (0)
Preterm birth (<37 weeks)	12	**1.27 (1.07, 1.49)**	12.0 (0.33)	35	**1.35 (1.20, 1.52)**	29.5 (0.05)	47	**1.32 (1.20, 1.45)**	24.3 (0.07)

Notes: The table above presents pooled estimates for the PMA studies alongside studies included in the most recently-published PregCOV-19 Living Systematic Review (May 2022) that meet eligibility criteria for the PMA study comparing pregnant people with and without SARS-CoV-2 infection but that were not successfully recruited to the PMA. We present these results for 7 common outcomes for this comparison group across the two studies: Intensive care unit admission; all-cause mortality; caesarean delivery; stillbirth; neonatal death; admission to a neonatal unit at birth; and preterm birth before 37 weeks gestation. These meta-analysis results follow the same methods described for the PMA.

1 PregCOV-19 Living Systematic Review studies incorporated in this analysis include those that 1) recruited a minimum of 25 cases of SARS-CoV-2 infection during pregnancy, 2) recruited a concurrent comparison group of uninfected pregnancies (i.e., excluding historical comparison cohorts, and 3) were not included in the PMA study analysis. These 53 studies include the following: Abedzadeh-Kalahroudi, 2021, Iran; Adhikari, 2020, USA; Afshar, 2020, USA; Anuk, 2021, Turkey; Beharier, 2021, Israel; Campbell, 2020, USA; Cardona-Pérez, 2021, Mexico; Celik, 2021, Turkey; Cuñarro-López, 2020, Spain; Debelenko, 2021, USA; Diaz-Corvillon, 2020, Chile; Dotters-Katz, 2020, USA; Edlow, 2020, USA; Egerup, 2020, Denmark; Elenga, 2021, French Guiana; Erol Koc, 2020, Turkey; Flaherman, 2020, USA; Gold, 2021, USA; Hcini, 2020, French Guiana; Izewski, 2021, USA; Jaiswala, 2021, India; Janssen, 2020, USA; Jering, 2021, USA; Levitan, 2021, USA; Liu, 2020, China; Liu, 2021, USA; Martinez-Perez, 2020, Spain; Maru, 2020, USA; McLaren, 2021, USA; Molenaar, 2021, USA; Money, 2021, Canada; Nayak, 2020, India; Ona, 2021, USA; Patberg, 2020, USA; Pineles, 2020, USA; Pirjani, 2020, Iran; Prabhu, 2020, USA; Rios-Silva, 2020, Mexico; Rosenbloom, 2021, USA; Ruggiero, 2020, Italy; Smithgall, 2020, USA; Soto-Torres, 2021, USA; Steffen, 2021, USA; Trahan, 2021, Canada; Tsatsaris, 2020, France; Vielma, 2020, Chile; Villar, 2021, multi-country study (includes 18 countries); Vouga, 2020, multi-country study (includes 16 countries); Wang, 2020, USA; Yadav, 2020, India; Yang, 2020, China; Zhang, 2020, USA; Zhang, 2021, USA.

2 Relative risks are calculated by pooling unadjusted relative risks from all participating studies with at least 1 adverse event for the given outcome using a DerSimonian-Laird random effects model meta-analysis. For any study with zero events in one arm (COVID-19 cases or COVID-negative comparisons), we used a continuity correction of 0.5.

3 The PMA outcome presented here is stillbirths occurring at or after 28 weeks gestational age per the WHO definition. For PregCOV-19 Studies, the estimates presented are as-reported by the study site and may span multiple different definitions of stillbirth (20-28 weeks).

4 The outcome "neonatal death" is reported by 9 participating studies in the PMA. However, most studies included in the PMA were not designed to follow-up neonates until 28 days after birth. The count of neonatal deaths is likely an underestimate.

5 The PMA outcome "NICU Admission at Birth" is defined as admission to the neonatal intensive care unit or the equivalent for all studies except for Crovetto, 2020, Spain, Cohort II, where the outcome also includes "admission to high-dependency care unit." The outcome for PregCOV-19 presented is defined as "admission to neonatal unit at birth."

Table S5. Sensitivity analysis comparing pooled absolute and relative risk of adverse outcomes using multiple definitions of stillbirth[1]

			Confirmed COVID-19 Case		COVID-19 Negative Comparison			
**Outcomes**	**N Studies**	**Included studies [2,3]**	**Events/Total**	**Pooled Risk[4] (95% CI)**	**Events/Total**	**Pooled Risk[4] (95% CI)**	**Relative Risk[5] (95% CI)**	**I^2% (p value)**
Stillbirths at or after 28 weeks gestation [6]	12	a b c d* e1 e2 f g h i j* k	14 / 1602	0.01 (0.00, 0.02)	64 / 10060	0.01 (0.00, 0.01)	1.08 (0.53, 2.16)	0 (0.97)
Stillbirths at or after 24 weeks gestation [7]	12	a b c d* e1 e2 f g h i j* k	21 / 1609	0.01 (0.0, 0.2)	84 / 10080	0.01 (0.0, 0.01)	0.92 (0.53, 1.59)	0 (0.99)
Stillbirths at or after 22 weeks gestation [8]	12	a b c d* e1 e2 f g h i j* k	24 / 1612	0.01 (0.0, 0.02)	99 / 10095	0.01 (0.01, 0.02)	0.89 (0.52, 1.51)	0 (0.99)
Stillbirths at or after 20 weeks gestation [9]	11	b c d e1 e2 f g h i j* k	25 / 1516	0.01 (0.00, 0.03)	94 / 7670	0.01 (0.01, 0.02)	0.88 (0.51, 1.50)	0 (0.97)

1 This analysis presents pooled absolute and relative risk of stillbirth based on four separate definitions by gestational age (at or after 28, 24, 22, or 20 weeks gestation). The fourth estimate (20 weeks gestation) does not Ahlberg et al. (2020, Sweden) because the study draws from a registry that does not include miscarriages (i.e., pregnancy loss before 22 weeks gestational age per the local definition).
2 Included studies for each estimate are categorized as follows: a) Ahlberg, 2020, Sweden; b) Akelo, Tippett Barr, 2021, Kenya; c) Bevilacqua, Laurita Longo 2020, Italy; d) Brandt, 2020, New Brunswick, USA; e1) Crovetto, 2020, Spain, Cohort I; e2) Crovetto, 2020, Spain, Cohort II; f) Kalafat, 2020, Turkey; g) Le Doare, 2021, Uganda; h) Nachega, 2021, Multi-country Africa; i) Nunes, 2021, South Africa; j) Poon, 2021, China-Hong Kong; k) Sakowicz, 2021, Chicago, USA
3 Asterisks (*) indicate there are zero total outcome events for a given study. These studies are not included in the "Events/Total" and pooled risk estimates.
4 Pooled absolute risks are calculated using Freeman-Tukey transformed proportions, pooled from all participating studies with at least 1 adverse event for the given outcome, using a DerSimonian-Laird random-effects inverse-variance model meta-analysis.
5 Relative risks are calculated by pooling unadjusted relative risks from all participating studies with at least 1 adverse event for the given outcome using a DerSimonian-Laird random effects model meta-analysis. For any study with zero events in one arm (COVID-19 cases or COVID-negative comparisons), we used a continuity correction of 0.5.
6 The outcome presented here is the definition used throughout the PMA study: stillbirths occurring at or after 28 weeks gestational age per the WHO definition.
7 The outcome presented here is stillbirths occurring at or after 24 weeks gestational age per the definition used by the UK National Health Service.
8 The outcome presented here is stillbirths occurring at or after 22 weeks gestational age per the definition used by the European Medicines Agency.
9 The outcome presented here is stillbirths occurring at or after 20 weeks gestational age per the definition used by the US Centers for Disease Control and Prevention.

Table S6. Detailed risk of bias assessment related to selection of the exposed and unexposed cohorts for individual studies

Study	Selection			
	**Representativeness of the exposed cohort**	**Selection of the non-exposed cohort**	**Ascertainment of exposure (COVID-19 Cases)**	**Ascertainment of control (COVID-negative comparison group)**
**Akelo 2021**	Population-based pregnancy surveillance<br>Universal screening at antenatal care, after enrollment (50 cases, 47.17%)<br>Universal screening at delivery (11 cases, 10.38%)<br>Universal screening at enrollment (32 cases, 30.19%)<br>Hospitalized for COVID-19 (1 cases diagnosed while hospitalized and 11 hospitalized for COVID-19 management, 11.32%)<br>Other COVID-19 testing for clinical concern (1 case, 0.94%)	Same cohort as exposed cohort	positive PCR test result	negative PCR test result
**Le Doare 2021**	Clinic-based pregnancy surveillance:<br>PCR screening at recruitment in early pregnancy (19 cases, 27.5%, includes 1 patient hospitalized for COVID-19)<br>Diagnosed with probable COVID-19 during pregnancy (50 cases, 72.5% includes 1 patient hospitalized for COVID-19)	Drawn from the same cohort of pregnancies as the exposed group, all non-exposed observations received a negative test during PCR screening at recruitment. These cases were followed up through clinic-based pregnancy surveillance, although repeated PCR testing was generally not available.	Positive PCR test result or meeting criteria for probable COVID-19 as per the WHO	Negative PCR and/or negative antibody test at recruitment<br>**AND**<br>did not meet WHO criteria for probable COVID-19 infection during pregnancy follow-up visits.
**Crovetto 2020, Cohort I**	Phase 1 (Initial recruitment from March-May 2020): Pregnant women at 10-16 weeks of gestation who provided blood sample for Down's syndrome screening were tested for SARS- CoV-2 antibodies (141 cases, 81.5%)<br>Phase 2: Follow-up testing at labor and delivery for all phase 1 participants using PCR; 32 cases, 18.5%)	Same cohort as exposed cohort	Tested positive for SARS-CoV-2 antibodies in early pregnancy<br>**OR**<br>PCR positive at delivery	Tested negative for SARS-CoV-2 antibodies in early pregnancy<br>**AND**<br>negative PCR test at delivery
**Poon 2021**	Testing for "clinical concern"<br>Universal testing for admission to the Labor & Delivery<br>Universal testing for any hospital admission	The first 2 patients who delivered following a confirmed COVID-19 case; if none, deliveries from the preceding day were selected.	positive PCR test result	negative serology test during routine Down syndrome screening (11-13 weeks' gestation) and again at delivery
**Crovetto 2020, Cohort II**	Universal testing at Labor & Delivery<br>(Apr-May 2020): Universal testing (antibody testing for all, PCR testing for some) at Labor & Delivery<br><br>Tested positive with PCR test only: 3 cases (1.7%)<br>Tested positive for antibody test only: 138 cases (78.4%)<br>Tested positive with both PCR and antibody test: 35 cases (19.9%)	Same cohort as exposed cohort	PCR positive test result, positive antibody test result, or positive PCR and antibody test at delivery	Negative antibody test at delivery or negative PCR and antibody test at delivery
**Bevilacqua 2020**	Testing for "clinical concern" (50 cases, 45.87%)<br>Universal testing for admission to the Labor & Delivery (19 cases, 17.43%)<br>Universal testing for hospital admission other than Labor & delivery, including pregnancy-related admission pre-term (40 cases, 36.70%)	Same cohort as exposed cohort	positive PCR test result	negative PCR test result

**Nachega 2021**	Symptomatic hospitalized women	Symptomatic hospitalized women	positive PCR test result	negative PCR test result
**Nunes 2021**	All women presenting for antenatal care (or admitted during pregnancy) with COVID-like symptoms (119 cases, 85.6%)<br>Screening of 10 asymptomatic women per day (19 cases, 13.7%)<br>Retested at labor & delivery after initial recruitment during antenatal care (1 case, 0.7%)	Same cohort as exposed cohort	positive PCR test result	PCR confirmed negative result
**Sakowicz 2020**	(March 2020-Feb2021): Testing for "clinical concern" (357 cases, 71%)<br><br>(Mar 2020-Feb 2021): Universal testing for admission to the Labor & Delivery (146 cases, 29%)	(April 2020 to May 2020): Universal testing for admission to the Labor & Delivery	Electronic health records (EHRs) indicating PCR positive test results	Electronic health records (EHRs) indicating negative PCR test result
**Ahlberg 2020**	Universal PCR testing for admission to the Labor & Delivery (142 cases, 91.02%)<br>Record of PCR or antibody positive test during antenatal care (14 cases, 8.97%)<br>Positive during pregnancy but negative upon admission (11 cases, 7.05%)<br>Positive antibody test not tested upon delivery (3 cases, 1.92%)	Universal PCR testing for admission to the Labor & Delivery	positive PCR test result<br>Record of PCR or antibody positive test during antenatal care	negative PCR test upon admission for delivery (no previous positive test)
**Kalafat 2020**	Asymptomatic pregnant women admitted for delivery (19 cases, 24.7%)<br>Symptomatic pregnant women evaluated for probable SARS-CoV-2 infection (58 cases, 75.3%)	Same cohort as exposed cohort	positive PCR test result	negative PCR test result
**Brandt 2020**	(Mar-Apr 2020): Testing for "clinical concern", recent travel, or exposure to known case (10 cases, 16.7%)<br>(Apr-Jun 2020): Universal testing for admission to the Labor & Delivery (50 cases, 83.3%)	Each COVID-19 case was matched to 2 controls by delivery date. Before April 10, controls were selected as the first 2 patients who delivered between 16.0- and 41.6-weeks' gestation on the same date as the cases if they were asymptomatic or had a negative COVID-19 test result. After April 10, controls were selected if they had a negative COVID-19 test result and delivered on the same date as the cases. On days with 2 or more cases, we identified the next 2 eligible controls as potential matches.	positive PCR test result	negative PCR test result

Table S7. Detailed risk of bias assessment related to outcome assessment for individual studies

Study	Outcome		
	Assessment of outcome	Adequacy of follow up of cohorts for pregnancy outcome	Adequacy of data completeness[1]
**Akelo 2021**	Clinical data were routinely collected in real time based on clinic and home visits	Number of pregnancies with pregnancy outcome known: 817 (52.4%)<br>Note: This is an ongoing cohort study and some missing pregnancy outcomes are because women have not yet delivered. The percentage of pregnancies with a recorded endpoint among those with expected due dates 4 weeks or more before the date data was shared is 83%.	a) Critical Care Indicators: Data excluded due to missingness >25%<br>b) Maternal Mortality & Morbidity: 0% Missing<br>c) Fetal & Neonatal Mortality & Morbidity: Neonatal follow up in progress still and thus excluded<br>d) Adverse Birth Outcomes: Missing 7% preterm, 9% birthweight, 21% SGA
**Le Doare 2021**	Clinical data were routinely collected in real time based on clinic visits	Number of pregnancies with pregnancy outcome known: 516 (97.0%)	a) Critical Care Indicators: Data not available<br>b) Maternal Mortality & Morbidity: <1% missing<br>c) Fetal & Neonatal Mortality & Morbidity: <1% missing<br>d) Adverse Birth Outcomes: < 2% missing
**Crovetto 2020, Cohort I**	All patient data were abstracted from the electronic medical records or hospital records	Number of pregnancies with pregnancy outcome known: 790 (85.8%)	a) Critical Care Indicators: 0% missing<br>b) Maternal Mortality & Morbidity: <4% missing for all, except PROM (excluded due to 36.5% missing outcome data)<br>c) Fetal & Neonatal Mortality & Morbidity: <1% missing, except NICU (excluded due to 39.4% missing outcome data)<br>d) Adverse Birth Outcomes: <3% missing
**Poon 2021**	Electronic health records and clinical report	Number of pregnancies with pregnancy outcome known: 152 (100%)	a) Critical Care Indicators: 0% missing<br>b) Maternal Mortality & Morbidity: 0% missing<br>c) Fetal & Neonatal Mortality & Morbidity: 0% missing<br>d) Adverse Birth Outcomes: 0% missing
**Crovetto 2020, Cohort II**	All patient data were abstracted from the electronic medical records or hospital records	Number of pregnancies with pregnancy outcome known: 1,304 (100%)	a) Critical Care Indicators: 0% missing<br>b) Maternal Mortality & Morbidity: 0% missing<br>c) Fetal & Neonatal Mortality & Morbidity: $\leq$ 2% missing<br>d) Adverse Birth Outcomes: < 1% missing
**Bevilacqua 2020**	Clinical data were routinely collected in real time in the patient's electronic medical records.	Number of pregnancies with pregnancy outcome known: 2464 (100%)	a) Critical Care Indicators: 0% missing<br>b) Maternal Mortality & Morbidity: 0% missing, except c-section (1.1%)<br>c) Fetal & Neonatal Mortality & Morbidity: 0% missing<br>d) Adverse Birth Outcomes: <1% missing

**Nachega 2021**	All patient data were abstracted from the patient hospital charts and logbooks	Number of pregnancies with pregnancy outcome known: 228 (51.6%)	a) Critical Care Indicators: <3%, except pneumonia (21.9%)<br>b) Maternal Mortality & Morbidity: <8%, except c-section (23%)<br>c) Fetal & Neonatal Mortality & Morbidity: <2%<br>d) Adverse Birth Outcomes: <20%
**Nunes 2021**	All patient data were abstracted from the hospital records or telephonic contact	Number of pregnancies with pregnancy outcome known: 746 (95.5%)	a) Critical Care Indicators: Data not available<br>b) Maternal Mortality & Morbidity: <1% missing; except placental abruption (> 99% missing, excluded)<br>c) Fetal & Neonatal Mortality & Morbidity: <1% missing (NICU admission not available)<br>d) Adverse Birth Outcomes: ≤5.2% missing
**Sakowicz 2020**	Electronic health records (EHRs) were reviewed for all pregnant women identified to have a SARS-CoV-2 test performed	Number of pregnancies with pregnancy outcome known: 1773 (100%)	a) Critical Care Indicators: <1% missing<br>b) Maternal Mortality & Morbidity: <1% missing<br>c) Fetal & Neonatal Mortality & Morbidity: 21.6% missing NICU, neonatal death (excluded due to high differential missingness between the exposed and non-exposed cohorts)<br>d) Adverse Birth Outcomes: <1% missing
**Ahlberg 2020**	Maternal and neonatal data were collected from the Swedish Pregnancy Register2 and medical records.	Number of pregnancies with pregnancy outcome known: 2682 (100%)	a) Critical Care Indicators: Data not available<br>b) Maternal Mortality & Morbidity: 0% missing, except haemorrhage (4.6%)<br>c) Fetal & Neonatal Mortality & Morbidity: 0% missing during observed period, but 28 day follow not part of study<br>d) Adverse Birth Outcomes: <1% missing
**Kalafat 2020**	All patient data were abstracted from the patient hospital charts, logbooks and electronic patient records.	Number of pregnancies with pregnancy outcome known: 351 (97.0%)	a) Critical Care Indicators: 0% missing<br>b) Maternal Mortality & Morbidity: 0% missing<br>c) Fetal & Neonatal Mortality & Morbidity: <1% missing<br>d) Adverse Birth Outcomes: <1 missing
**Brandt 2020**	All patient data were abstracted from the electronic medical records	Number of pregnancies with pregnancy outcome known: 162 (100%)	a) Critical Care Indicators: 0% missing<br>b) Maternal Mortality & Morbidity: 0% missing<br>c) Fetal & Neonatal Mortality & Morbidity: 0% missing<br>d) Adverse Birth Outcomes: <1% missing

1 Additional information on adequacy of data completeness for each individual outcome is presented in Table S8.

Table S8: Description of Follow-up by Study and Review of Missing Data by Outcome [1]

Study (Author Year, Site)	Overall Follow-up		Missing Data by Outcome: among all pregnancies			
	Number of pregnancies identified	Number of pregnancies with a recorded endpoint (%)	ICU admission	Ventilation	Critical care	Pneumonia
Akelo, Tippett Barr 2021, Kenya [2]	1560	817 (52.4%) [2]	99.4%*	99.4%*	99.4%*	99.4%*
Le Doare, 2021, Uganda [3]	532	516 (97.0%)[3]	--	--	--	--
Crovetto 2020, Spain, Cohort I	921	790 (85.8%)	0.0%	0.0%	0.0%	0.0%
Poon 2021, China-Hong Kong	152	152 (100%)	0.0%	0.0%	0.0%	0.0%
Crovetto 2020, Spain, Cohort II	1,304	1,304 (100%)	0.0%	0.0%	0.0%	0.0%
Bevilacqua, Laurita Longo 2020, Italy	2465	2464 (100%)	0.0%	0.0%	0.0%	0.0%
Nachega 2021, Afrehealth	442	228 (51.6%)	0.0%	2.7%	2.0%	21.9%
Nunes 2021, South Africa	781	746 (95.5%)	--	--	--	--
Sakowicz, 2021, USA [4]	1773	1773 (100%)	0.4%	--	--	--
Ahlberg, 2020, Sweden	2682	2682 (100%)	--	--	--	--
Kalafat 2020, Turkey	362	351 (97.0%)	0.0%	0.0%	0.0%	0.0%
Brandt 2020, USA	162	162 (100%)	0.0%	0.0%	0.00%	--

[1] For each outcome, this table shows the percentage of missing data for each outcome. For our meta-analysis, we excluded any outcome that was more than 25% missing. These excluded outcomes are indicated with an asterisk. Outcomes that were not collected for a study are noted using the "--" symbol.

[2] The ANCOV Kenya study (Akelo, Barr, 2021) is an ongoing cohort study conducting population-level surveillance and some missing pregnancy outcomes can be accounted for by pregnant women who have been identified for the study but have not yet delivered. Among the 885 pregnancies with an expected due date 4 weeks or more before the date data was shared (August 19, 2021), 83% have completed pregnancy follow up.

[3] The PREPARE Uganda study (Le Doare 2021) is an ongoing cohort study and some missing pregnancy outcomes can be accounted for by pregnant women who have been identified for the study but have not yet delivered. The percentage of pregnancies with a recorded endpoint among those with expected due dates 4 weeks or more before the date data was most recently updated (October 31, 2021) is 97.4%, with 16 observations missing pregnancy outcome out of 530 pregnancies.

[4] We excluded the Sakowicz study (2021, USA) from the pooled estimates for NICU admission and neonatal death because of differential missingness across the COVID-19 cases in pregnancy and COVID-negative comparison group. Neonatal death was 68% missing for COVID-19 cases in pregnancy, but only 3% missing for COVID-negative pregnancies. NICU Admission was 68% missing for COVID-19 cases in pregnancy, but only 2% missing for COVID-negative comparison pregnancies.

Table S8: Description of Follow-up by Study and Review of Missing Data by Outcome [1], continued

Study (Author Year, Site)	Missing Data by Outcome: among all completed pregnancies (with a recorded pregnancy endpoint)										
					Hypertensive disorders of pregnancy						
	Maternal death	PROM	Haemorrhage	Placental abruption	Diagnosed at or after COVID-19	Diagnosed at any time	Preeclampsia or Eclampsia	Thromboembolic disease	Preterm labor	Preterm labor - COVID onset <37w	Cesarian section
Akelo, Tippett Barr 2021, Kenya [2]	--	--	--	--	0.0%	0.0%	0.0%	--	--	--	--
Le Doare, 2021, Uganda [3]	0.0%	0.0%	0.0%	--	--	0.0%	0.0%	0.0%	0.0%	0.0%	0.0%
Crovetto 2020, Spain, Cohort I	0.0%	36.5%*	--	--	--	0.0%	0.0%	0.0%	3.9%	--	0.8%
Poon 2021, China-Hong Kong	0.0%	0.0%	--	0.0%	0.0%	0.0%	0.0%	0.0%	0.0%	0.0%	0.0%
Crovetto 2020, Spain, Cohort II	0.0%	0.0%	--	--	--	0.0%	0.0%	0.0%	0.0%	--	0.0%
Bevilacqua, Laurita Longo 2020, Italy	0.0%	--	0.0%	--	--	0.0%	0.0%	0.0%	0.0%	0.0%	1.1%
Nachega 2021, Afrehealth	0.4%	7.5%	7.0%	6.6%	--	3.5%	5.7%	--	26.3%*	6.9%	22.9%
Nunes 2021, South Africa	0.7%	0.0%	0.0%	99.5%*	--	0.0%	0.0%	0.0%	0.0%	0.0%	0.1%
Sakowicz, 2021, USA [4]	--	--	0.2%	0.0%	--	0.4%	0.4%	--	--	--	0.0%
Ahlberg, 2020, Sweden	0.0%	0.0%	4.4%	0.0%	0.0%	0.0%	0.0%	0.0%	--	--	0.0%
Kalafat 2020, Turkey	0.0%	--	--	0.0%	--	--	--	--	--	--	--
Brandt 2020, USA	0.0%	--	--	--	--	--	--	0.0%	--	--	0.0%

[1] For each outcome, this table shows the percentage of missing data for each outcome. For our meta-analysis, we excluded any outcome that was more than 25% missing. These excluded outcomes are indicated with an asterisk. Outcomes that were not collected for a study are noted using the "--" symbol.

[2] The ANCOV Kenya study (Akelo, Barr, 2021) is an ongoing cohort study conducting population-level surveillance and some missing pregnancy outcomes can be accounted for by pregnant women who have been identified for the study but have not yet delivered. Among the 885 pregnancies with an expected due date 4 weeks or more before the date data was shared (August 19, 2021), 83% have completed pregnancy follow up.

[3] The PREPARE Uganda study (Le Doare 2021) is an ongoing cohort study and some missing pregnancy outcomes can be accounted for by pregnant women who have been identified for the study but have not yet delivered. The percentage of pregnancies with a recorded endpoint among those with expected due dates 4 weeks or more before the date data was most recently updated (October 31, 2021) is 97.4%, with 16 observations missing pregnancy outcome out of 530 pregnancies.

[4] We excluded the Sakowicz study (2021, USA) from the pooled estimates for NICU admission and neonatal death because of differential missingness across the COVID-19 cases in pregnancy and COVID-negative comparison group. Neonatal death was 68% missing for COVID-19 cases in pregnancy, but only 3% missing for COVID-negative pregnancies. NICU Admission was 68% missing for COVID-19 cases in pregnancy, but only 2% missing for COVID-negative comparison pregnancies.

Table S8: Description of Follow-up by Study and Review of Missing Data by Outcome [1]**,** continued

Study (Author Year, Site)	Missing Data by Outcome: among all births or livebirths (with a recorded pregnancy endpoint)							
	**Stillbirth**	**Perinatal death**	**Neonatal death**	**NICU Admission at birth**	**Low birthweight (<2500g)**	**Small for gestational age (10th)**	**Preterm birth (<37 weeks)**	**Preterm birth (<37 weeks) - COVID onset <37w**
Akelo, Tipett Barr 2021, Kenya [2]	0.1%	26%*	26.5%*	--	8.7%	20.9%	7.1%	6.7%
Le Doare, 2021, Uganda [3]	0.2%	0.4%	0.0%	0.2%	0.0%	1.7%	0.8%	0.8%
Crovetto 2020, Spain, Cohort I	0.0%	0.4%	0.4%	39.4%*	1.3%	2.9%	1.2%	--
Poon 2021, China-Hong Kong	0.0%	0.0%	0.0%	0.0%	0.0%	0.0%	0.0%	0.0%
Crovetto 2020, Spain, Cohort II	0.0%	0.0%	0.0%	2.0%	0.0%	0.3%	0.0%	--
Bevilacqua, Laurita Longo 2020, Italy	0.0%	0.0%	0.0%	0.0%	0.1%	0.2%	0.0%	0.0%
Nachega 2021, Afrehealth	1.5%	--	0.0%	--	14.2%	19.1%	12.6%	14.9%
Nunes 2021, South Africa	0.1%	0.5%	0.4%	--	3.0%	5.2%	0.4%	0.4%
Sakowicz, 2021, USA [4]	0.0%	--	21.6%[4]	21.0% [4]	0.1%	0.3%	0.1%	0.0%
Ahlberg, 2020, Sweden	0.0%	0.0%	0.0%	0.0%	0.4%	0.7%	0.0%	--
Kalafat 2020, Turkey	0.0%	0.3%	0.3%	0.6%	0.0%	0.0%	0.0%	0.0%
Brandt 2020, USA	0.0%	0.0%	0.0%	0.0%	0.0%	0.6%	0.0%	0.0%

[1] For each outcome, this table shows the percentage of missing data for each outcome. For our meta-analysis, we excluded any outcome that was more than 25% missing. These excluded outcomes are indicated with an asterisk. Outcomes that were not collected for a study are noted using the "--" symbol.
[2] The ANCOV Kenya study (Akelo, Barr, 2021) is an ongoing cohort study conducting population-level surveillance and some missing pregnancy outcomes can be accounted for by pregnant women who have been identified for the study but have not yet delivered. Among the 885 pregnancies with an expected due date 4 weeks or more before the date data was shared (August 19, 2021), 83% have completed pregnancy follow up.
[3] The PREPARE Uganda study (Le Doare 2021) is an ongoing cohort study and some missing pregnancy outcomes can be accounted for by pregnant women who have been identified for the study but have not yet delivered. The percentage of pregnancies with a recorded endpoint among those with expected due dates 4 weeks or more before the date data was most recently updated (October 31, 2021) is 97.4%, with 16 observations missing pregnancy outcome out of 530 pregnancies.
[4] We excluded the Sakowicz study (2021, USA) from the pooled estimates for NICU admission and neonatal death because of differential missingness across the COVID-19 cases in pregnancy and COVID-negative comparison group. Neonatal death was 68% missing for COVID-19 cases in pregnancy, but only 3% missing for COVID-negative pregnancies. NICU Admission was 68% missing for COVID-19 cases in pregnancy, but only 2% missing for COVID-negative comparison pregnancies.
